# Health risk behaviours, mental health and HbA1c: an overview of reviews of observational studies

**DOI:** 10.1136/bmjopen-2024-092657

**Published:** 2025-11-16

**Authors:** Soumya Mazumdar, Nerea Almeda, Nasser Bagheri, Mark Daniel, Hossein Tabatabaei Jafari, Gweneth Leigh, Diego Diaz Milanes, Luis Salvador-Carulla

**Affiliations:** 1 Health Research Institute, University of Canberra Faculty of Health, Bruce, Australian Capital Territory, Australia; 2 Universidad Loyola Andalucía, Cordoba, Spain; 3 Dasman Diabetes Institute, Kuwait City, Kuwait; 4 Universidad Loyola Andalucia - Campus de Sevilla, Sevilla, Spain

**Keywords:** MENTAL HEALTH, Diabetes Mellitus, Type 2, Behavior, PUBLIC HEALTH, Observational Study, Review

## Abstract

**Abstract:**

**Objectives:**

To implement an overview of reviews that discuss the current state of syntheses (such as systematic reviews) of only observational studies on health risk behaviours (HRBs), including smoking, alcohol intake, poor sleep, poor quality diet, common mental health problems (depression and anxiety), and glycated haemoglobin (HbA1c), while excluding synthesis of clinical trials.

**Design:**

Overview of reviews or umbrella review following Preferred Reporting Items for Overviews of Reviews (PRIOR) guidelines.

**Data sources:**

PubMed, Scopus, Web of Science, PsycINFO-PsychArticles and Epistemonikos, searched from January 2013 to 30 June 2025.

**Eligibility criteria:**

We included systematic reviews and meta-analyses of observational studies that assessed the relationship between HRBs—including smoking, alcohol intake, poor sleep, poor quality diet, physical activity and common mental health problems such as depression and anxiety—and HbA1c. Reviews of clinical trials were excluded.

**Data extraction and synthesis:**

We synthesised systematic reviews and meta-analyses on the above topic from five databases following the PRIOR protocol. Two independent reviewers screened titles, abstracts and full texts using standardised methods. Data extracted included study design, exposures, outcomes and population characteristics. Risk of bias was assessed using the AMSTAR-2 tool. Overlap across reviews was evaluated using the corrected covered area metric.

**Results:**

Eight systematic reviews were included in the final synthesis, encompassing a total sample size of around 307 019 individuals. The study highlights a significant paucity of systematic reviews of observational studies in this area, with no reviews on alcohol and exercise. The existing evidence on poor sleep, poor quality diet and smoking points towards these HRBs leading to worse HbA1c. A bidirectional relationship was found between depression and HbA1c.

**Conclusions:**

This umbrella review highlights the significant association between HbA1c and key health risk factors underscoring the importance of observational studies, highlighting their ability to capture real-world conditions and complex interactions. While in agreement with existing study designs, this review provides convergent evidence of the critical role of HRBs in managing HbA1c levels.

**STRENGTHS AND LIMITATIONS OF THIS STUDY**

- A Preferred Reporting Items for Overviews of Reviews guideline-based umbrella review exclusively of observational studies.
- Risk of bias was assessed using the AMSTAR-2 tool, with high inter-rater agreement.
- Small number of eligible reviews and heterogeneity in study designs.
- Reviews on key exposures such as alcohol, physical activity and anxiety were lacking, highlighting gaps in the existing literature.

## Introduction

 A large body of literature has been underscoring the effect of ‘diabetogenic’ and ‘obesogenic’ social and built contextual environments on health behaviours and mental health.^1^,^3^ Such environments may influence health risk behaviours (HRBs) or behaviours that increase the likelihood of adverse physical, social or psychological consequences.^4^ HRBs such as sleep, physical activity/sedentarism and diet may be influenced by the contextual environment through various complex pathways and lead to various chronic non-communicable diseases including diabetes and common mental health problems^5^—depression and anxiety.^6^ In this regard, contextual environments encompass the broader social and environmental conditions that shape health and illness and are essential to understanding the complex, multifactorial nature of health outcome.^7^

Contextual factors that affect health manifest through complex pathways characterised as volatile, uncertain, complex and ambiguous (VUCA).^8^ While randomised controlled trials (RCTs) are commonly used to investigate the effects of HRBs on outcomes,^9^ they often lack the capacity to capture the intricate interactions between individual traits and contextual factors such as the built or social environment due to their strictly controlled settings, constrained external validity and limited generalisability to diverse populations and real-life complex and emergent VUCA scenarios.^10^,^14^ While individually randomised RCTs randomise across individual levels and may investigate subgroup effects through stratification, it is difficult to randomise across contextual environments without recourse to complex designs such as cluster randomised trials. In contrast, observational studies can explore how individual characteristics interact with contextual factors, adding valuable information to that provided by RCTs. For example, an observational study may examine the effect of exercise on weight loss for people living in walkable neighbourhoods versus car-dependent suburbs. While it is true that RCTs—particularly those designed with an effectiveness focus—can attempt to emulate real-world conditions and may stratify analyses by contextual variables such as neighbourhood type, such designs still face practical and ethical constraints in randomising across diverse environmental settings. Moreover, the degree of control in RCTs varies along a spectrum from tightly controlled efficacy trials to more pragmatic effectiveness trials, with the latter aiming to enhance external validity but still often falling short of capturing the full complexity of real-world, heterogeneous environments. Observational studies, by contrast, are inherently suited to capturing these complexities. They operate in fully naturalistic settings, allowing for the study of diverse populations and contextual interactions without the constraints of intervention protocols or randomisation. Unlike pragmatic trials, which still require structured intervention delivery and monitoring, observational studies can investigate exposures that are unethical or impractical to randomise. They also allow for larger sample sizes, lower costs and longer follow-up durations, making them particularly valuable for studying chronic conditions and rare outcomes. Furthermore, modern analytical techniques, such as propensity score matching and Mendelian Randomisation, enhance the causal inference capabilities of observational designs, offering complementary insights to those derived from RCTs.^15 16^

In addition, when the intervention is something such as lifestyle change or exercise, RCTs are subject to the Hawthorne effect.^17^ This means that participants could modify their attitudes or behaviours because of their awareness of being observed. Finally, there may be situations and circumstances where implementing an RCT may be unethical or otherwise infeasible. Indeed, while some RCT designs, such as cluster randomised designs, can incorporate context to an extent, observational studies that account for confounding and bias are better positioned to account for the Hawthorne effect and the great multitude of heterogenous contexts and populations, across which it may be difficult or unethical to define clusters and randomise.

One solution to the above issues may be to incorporate evidence from observational studies, alongside other sources of evidence, including RCTs. This agrees with an increasing acceptance of the need to move away from a unidimensional evidence-based medicine pyramid-based, RCT-dominated approach towards knowledge generation. Instead, many forms of knowledge sources such as observational studies, contextual evidence, cultural norms, expert insights and consumer experiences, in addition to RCTs, could support policy development and implementation or support evidence-informed decisions.^18 19^ Evidence-informed decision-making reflects a shift away from rigid hierarchies of evidence towards a more inclusive approach that values diverse types of knowledge. According to the WHO, this approach emphasises identifying, appraising and mobilising the best available evidence—including qualitative and contextual data—to inform policies and programmes.^20^

As discussed, observational studies are likely to incorporate the real-life, heterogenous contexts and populations in which HRBs and their outcomes are more likely to manifest.^21^ Observational studies allow for testing interventions in specific local contexts, inclusion of local expert knowledge and provide factual knowledge for policy making.^20^ Also, according to the National Institute for Healthcare Excellence real-world evidence framework, observational studies can relatively easily help fill gaps in the evidence base.^14^ While observational studies may have issues with confounding, reverse causation and other potential biases, when triangulated with other forms of knowledge, this can inform collective decision-making through consilience to drive health policy and research.^22^,^24^ When appropriately designed and implemented, observational studies are not more likely to provide biased estimates relative to RCTs.^25^ Finally, ‘emulated target trials’ use observational databases to answer causal questions about the comparative effects of interventions when randomised trials are unavailable or not feasible. The Transparent Reporting of Observational Studies Emulating a Target Trial guideline has been developed to improve reporting transparency of these studies.^26^ In summary, experimental and observational studies are both required and important in the framing of scientific literature.^22^ Thus, there is increasing acceptance that both experimental and observational studies are important in the framing of scientific knowledge.

Another issue of importance to the literature of HRBs is the heterogeneity of measured outcomes that makes cross-study comparisons across a wide range of downstream outcomes difficult.^27^ One option may be to measure specific biomarkers which, when elevated or suppressed, indicate the presence of a range of diseases and conditions. For example, the biomarker salivary cortisol has been associated with exposure to various forms of stress, obesity and hypertension.^28^ Another important biomarker is glycated haemoglobin (HbA1c), which has been associated with a number of conditions such as diabetes, cardiovascular disease and even mental disorders.^6^ In this regard, mental health conditions are of specific interest, because of reported bidirectional relationships with HbA1c.^6^ HbA1c levels and other cardiometabolic indicators may become abnormal through a number of different pathways, some of which include various socio-environmental and individual level risk factors.^29^ Also, indicators such as HbA1c form one approach to measuring ‘Allostatic Load’ or the extent of wear and tear in the human body resulting from disease or HRBs.^30^ In addition to the use of a single indicator such as HbA1c to measure adverse outcomes, the myriad of HRBs and NCDs can also be organised into coherent taxonomies, to facilitate appropriate synthesis within studies, and allow cross-study comparisons.^31^

Thus, given that observational studies investigate relationships in naturalistic settings and offer allowance for contextual interactions in a safe and ethical manner, the literature on lifestyles and biomarkers could benefit from a synthesis of observational studies of the examining associations, and causal estimates^26^ of HRBs and mental health disorders on a biomarker such as HbA1c. While there are a few systematic reviews in this area,^32^ there remains scope for an overview of reviews, a synthesis of reviews, or an ‘umbrella review’^33^ of the various systematic reviews and meta-analyses of the relationship between HbA1c and HRBs. While existing guidelines suggest the use of ‘overview of reviews’ term,^34^ we use the more concise ‘umbrella review’ term in most of this document, except the title. An umbrella review can provide guidance on this topic, and on synergistic and contradictory research. It can also investigate whether reviews exploring the same research question independently arrive at the same answer, in addition to exposing gaps in the existing evidence base.^33^ To contrast systematic and umbrella reviews, while systematic reviews are rigorous syntheses of primary research studies focused on a specific question, umbrella reviews synthesise findings from multiple systematic reviews and meta-analyses, offering a high-level summary across related topics. While systematic reviews examine individual studies, umbrella reviews evaluate the consistency, gaps and convergence of evidence across existing reviews. This study aims (as discussed in detail in the next section) to assess the broader landscape of observational evidence on HbA1c and HRBs, rather than re-analysing primary data. As such, umbrella reviews often adopt narrower search terms than systematic reviews to maintain specificity and avoid redundancy, as they synthesise existing reviews rather than primary studies. A broader search may increase sensitivity but risks including overlapping or less relevant reviews, complicating synthesis.^35^

### Aims of the study

To address the above issues, we examined the following question through an umbrella review of systematic reviews and meta-analyses ‘What is the state of evidence in observational studies regarding the relationship between HRBs, mental health and HbA1c?’ We chose six key health risk factors: mental health conditions, sleep characteristics, diet, physical activity, alcohol and smoking that, in separate studies, have been found to be associated with HbA1c.^30 36^ These factors were chosen based on previous research on the taxonomy of health-related habits and lifestyles (eVITAL),^31 37^ and analysis of the relationships between local characteristics and cardiometabolic health.^29^ The previously published eVITAL taxonomy provides a structured and validated framework for categorising lifestyle and behavioural health domains, ensuring conceptual clarity and consistency across studies. Its use enhances the reproducibility and comprehensiveness of search strategies by aligning terms with well-defined constructs.^31 37^ Our overall aim was to synthesise existing systematic reviews and meta-analyses rather than conduct an exhaustive primary-level search. Therefore, we prioritised a focused and conceptually coherent search strategy to ensure feasibility, manageability and alignment with our predefined framework.

## Study design and methods

### Study design

Our umbrella review of systematic reviews and meta-analyses on the association among common mental health conditions, sleep characteristics, diet, physical activity, alcohol and smoking, and HbA1c follows the Preferred Reporting Items for Overviews of Reviews statement to the extent feasible.^34^ The protocol of this study was registered on PROSPERO on 2 October 2023 (PROSPERO ID: Suppressed for Peer Review). The process was supported by a research librarian.

### Information source and search strategy

The search was implemented on PubMed, Scopus, Web of Science (list of databases provided in online supplemental appendix A), PsycINFO-PsychArticles and Epistemonikos, from January 2013 to 30 June 2025 to assess relatively contemporary literature on the topic. The search was done in two stages, with the first one up until September 2024, and later updated to June 2025. Additionally, the reference lists of included articles were reviewed manually for any additional articles.

The search strategy comprised a combination of terms selected from the controlled vocabulary with free-text terms based on the six HRBs, mental health disorders and HbA1c. While the search syntax provided below is for the PubMed search, the same search strategy was used for other databases as well (online supplemental appendix A). The search is as follows:

Systematic[sb] AND ((“Sleep disorder*”[Title/Abstract] OR “Sleep wake disorder*”[Title/Abstract] OR “Sleep deprivation”[Title/Abstract] OR “Sleep arousal”[Title/Abstract] OR “Insomnia”[Title/Abstract] OR “Obstructive sleep apnea”[Title/Abstract] OR Anxiety[Title/Abstract] OR “Mood disorder”[Title/Abstract] OR Stress[Title/Abstract] OR “Psychological distress”[Title/Abstract] OR “depressive disorder”[MeSH Terms] OR depress*[Title/Abstract] OR “Depressive disorder”[Title/Abstract] OR “Depressive symptom”[Title/Abstract] OR “Dysthymic disorder”[Title/Abstract] OR “alcohol”[Title/Abstract] OR “smoking”[Title/Abstract] OR “diet”[Title/Abstract] OR “Physical activity”[Title/Abstract] OR “sedentarism”[Title/Abstract]) AND (HbA1c[Title/Abstract] OR “Hemoglobin A1C”[Title/Abstract] OR “Glycated Hemoglobin”[Title/Abstract])). The search strategy was performed by two independent researchers (SM and GL). Terms included in the sb filter in PubMed are provided in Appendix A.

### Eligibility criteria

The rationale for inclusion criteria was to facilitate a comprehensive analysis of all systematic reviews and meta-analyses of observational studies that assessed the relationship between mental health, HRBs as discussed previously, and HbA1c. Following the population, environment, comparator and outcomes (PECO) tool,^38^ we included general population and population with type 2 diabetes (P), various HRBs and mental health conditions (E), and HbA1c as outcome (O), with the comparator (C) being inapplicable. For the initial search, no restrictions were imposed on language or study setting.

### Study selection

Two reviewers (SM and GL) independently screened the studies in two phases to assess the eligibility. Initially, abstracts and titles were screened, followed by full-text screening for eligibility. Disagreements were resolved by consensus between the two reviewers. The overlap of populations, exposures and outcomes in the included studies was noted. Overlap of studies across the reviews was estimated using the corrected covered area (CCA) metric.^39^ The CCA is a quantitative measure used to assess the degree of overlap among primary studies included in multiple systematic reviews. It is particularly useful in umbrella reviews or overviews of reviews to evaluate redundancy and ensure that findings are not disproportionately influenced by the same underlying studies. The CCA is calculated by comparing the number of repeated studies across reviews to the total number of unique studies, adjusted for the number of reviews. A CCA value of 0%–5% indicates slight overlap, 6%–10% moderate, 11%–15% high and >15% very high overlap.^39 40^

### Data collection process

The two previously mentioned reviewers independently extracted all relevant data points into a spreadsheet specifically created for this study, which included all citation information, study design, region of study, the exposure and outcome, population characteristics and quality assessment implemented. To ensure consistency, the eVITAL taxonomy introduced earlier was used to code the HRBs and mental health conditions.^37^ Key relationships and outcomes of the studies were noted. Mean differences with 95% CIs were extracted from meta-analyses in addition to the total number of studies and total population included in each review. Also, unless otherwise stated, all studies report HbA1c as percentage (% units).

Risk of bias in the included systematic reviews and meta-analyses was assessed by two independent researchers (SM and GL) using the ‘A Measurement Tool to Assess Systematic Reviews’ or AMSTAR tool.^41^ Disagreements were resolved through discussion.

### Data and resource availability

All data generated or analysed during this study are included in the published article (and its online supplemental file).

### Patient and public involvement

Patients and the public were not involved.

## Results

### Further study selection

The initial search resulted in 2117 potentially relevant records (online supplemental material, Fig, S.1, appendix A). This comprised 541 from PubMed, 644 from Scopus, 517 from Web of Science, 134 from PsycINFO-PsychArticles and 281 from Epistemonikos. Of the 2117 records identified, 1463 were duplicates, which were indexed in more than one database and were excluded. Deduplication was done in EndNote.^42^ Of the remaining 654 records, 40 were excluded as they were either not meta-analyses or systematic reviews, or involved non-human studies, and 606 were excluded for reasons inclusive of not exclusively based on observational studies (606), and one that was not in English. Of the 606 studies, 467 or 77% involved RCTs. Finally, eight reviews were included.^632 43^,^48^ No additional articles were identified from scanning reference lists.

### Study characteristics

The eight reviews represented a total sample size of around 307 019 individuals and approximately 142 primary studies that investigated HbA1c as an outcome. There was a low level of overlap among the included articles among the eight systematic reviews with a CCA metric of less than 2%. The number of study subjects ranged from 3683 to 87 593 individuals. Various age ranges were reported in the studies with minimum age being between 15 and 37 and maximum age being between 53 and 89. While various countries were represented in the reviews, the majority of studies were from the European continent (four reviews), followed by the USA (two reviews).

Six reviews implemented a meta-analysis, while one review implemented only a systematic review^48^ and another, Zhu *et al*^47^, implemented an integrative review following a systematic review of the literature. While most of the studies included in the above reviews were cross-sectional, some^48 49^ also included studies with longitudinal designs. Four reviews focused on studies investigating individuals with type 2 diabetes mellitus (T2DM), and one study included individuals with type 1 diabetes mellitus or T2DM. Other reviews investigated studies that involved a mix of individuals with and without depression,^49^ healthy individuals who used to smoke or healthy individuals who currently smoke,^46^ and a review that included studies investigating both clinic and non-clinic populations.^45^ Note that the reviewed studies indicate the term ‘depression’ to indicate all types of depression and depressive symptoms, and we adopt this terminology throughout the document to remain consistent. The studies are summarised in table 1.

**Table 1:** Synthesised studies

Authors	Study design	Most common design of included studies	Outcome*	Exposure*	Statistic	Mean difference and 95% CI	Total sample (studies)	Total sample (population)
Beran *et al*^6^	Systematic review and meta-analysis	Longitudinal	v.6.5. Glycated haemoglobin	v.3.4.2. Depression	Correlation	0.07 (0.03 to 0.12)	6	3683
Beran *et al*^6^	Systematic review and meta-analysis	Longitudinal	v.3.4.2. Depression	v.6.5. Glycated haemoglobin	Correlation	(,)	5	45 110
Genis-Mendoza *et al*^43^	Systematic review and meta-analysis	Cross-sectional	v.6.5. Glycated haemoglobin	v.3.4.2. Depression	Mean difference	0.18 (0.12 to 0.29)	34	68 398
Kar *et al*^32^	Systematic review and meta-analysis	Cross-sectional	v.6.5. Glycated haemoglobin	u.s.3. Nicotine	Mean difference	−0.61% (−0.33 to 0.88)	10	87 593
Koopman *et al*^44^	Systematic review and meta-analysis	Cross-sectional	s.1.3. Other sleep-related behaviours	v.6.5. Glycated haemoglobin	Mean difference	0.23 (0.1 to 0.4)	14	17 067
Lee *et al*^45^	Systematic review and meta-analysis	Cross-sectional	v.6.5. Glycated haemoglobin	s.1.1. Sleep schedule	Mean difference	0.23 (0.1 to 0.36)	8	29 649
Lee *et al*^45^	Systematic review and meta-analysis	Cross-sectional	v.6.5. Glycated haemoglobin	s.1.2. Sleep quality	Mean difference	0.35 (0.12 to 0.28)	15	
Sepandi *et al*^48^	Systematic review	Cross-sectional	v.6.5. Glycated haemoglobin	d.2.5. Quality of diet	NA	NA	6	6337
Soulimane *et al*^46^	Systematic review and meta-analysis	Cross-sectional	v.6.5. Glycated haemoglobin	u.s.3. Nicotine	Mean difference		14	35 425
Zhu *et al*^47^	Integrative review	Cross-sectional	v.6.5. Glycated haemoglobin	s.1.2. Sleep quality	NA	NA	22	13 757

* The alphanumeric codes beside outcome and exposure represent specific eVITAL^31 37^ lifestyle and biometric category numbers.

HbA1c, glycated haemoglobin; NA, not applicable.

The exposures that were investigated in the eight reviews included depression, nicotine, quality of diet, sleep schedule and sleep quality, all of which map to specific eVITAL categories. No systematic reviews of observational studies were identified that focused on examining alcohol intake, exercise or anxiety. The associations identified between these exposures and HbA1c are summarised next.

### Associations identified 

**Figure 1:**
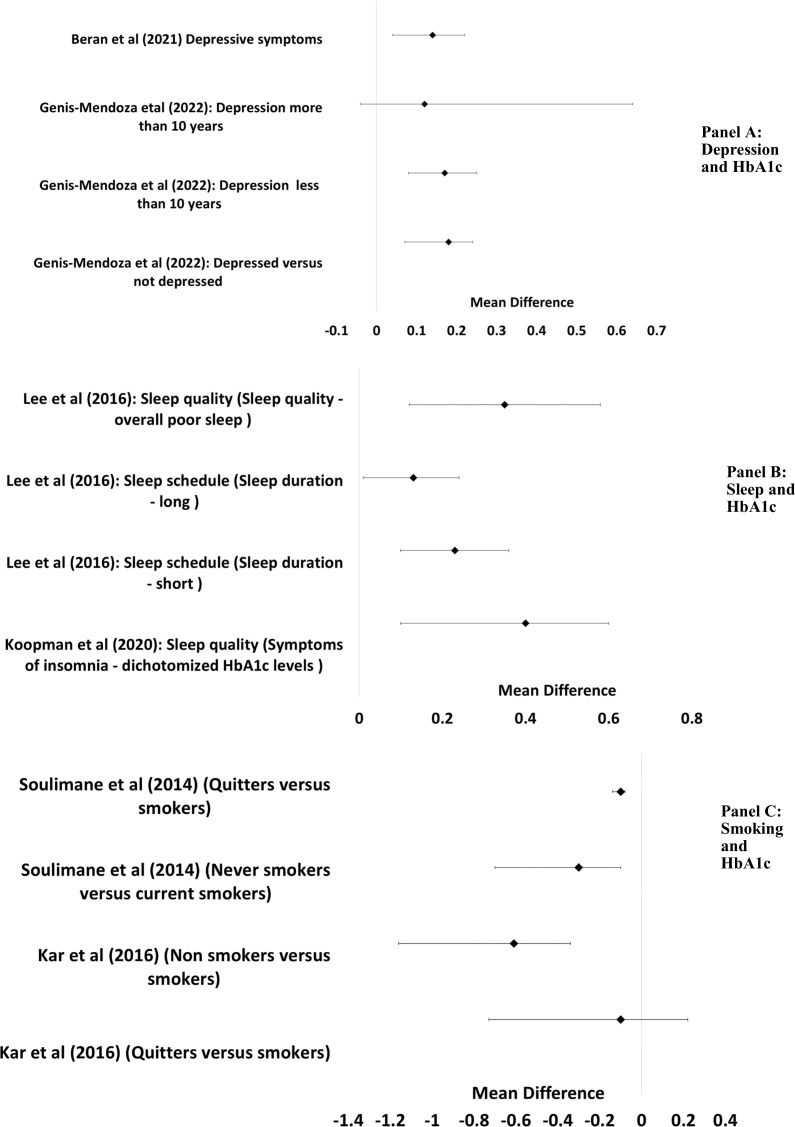
Relationship between depression, sleep, smoking and HbA1c. Reporting unit is in %. HbA1c, glycated haemoglobin.

A bidirectional longitudinal relationship between depressive symptoms and HbA1c levels in individuals with T2DM was identified.^49^ Higher baseline depressive symptoms were associated with subsequent increases in HbA1c (partial r=0.07, 95% CI 0.03 to 0.12), while higher baseline HbA1c was linked to an 18% increased risk of developing depression (OR=1.18, 95% CI 1.12 to 1.25).^49^ Complementing these findings, another study showed that individuals with T2DM and depression had significantly higher HbA1c levels compared with those without depression (Cohen’s d=0.18, 95% CI 0.12 to 0.29).^43^ This association remained consistent in subgroups taking hypoglycaemic medications (d=0.20), with less than 10 years of disease duration (d=0.17) and with diabetes-related complications (d=0.17). Together, these findings underscore a complex, bidirectional relationship between glycaemic control and depression in T2DM (figure 1, Panel A).

Sleep was found to be associated with HbA1c. Across three reviews, sleep disturbances were found to be highly prevalent in T2DM, with insomnia symptoms affecting up to 39% (34%–44%) of the population with T2DM.^44 45 47^ Both sleep duration and quality influence HbA1c levels, with poor glycaemic control potentially worsening sleep disturbances. Thus, patients with T2DM with insomnia symptoms have worse HbA1c (0.23% mean difference). Additionally, both short and long sleep durations were associated with elevated HbA1c levels—0.23% and 0.13%,^45^ respectively—suggesting a U-shaped relationship between sleep duration and glycaemic control.^45^ The reviews suggest various mechanisms linking sleep to glycaemic control, including hormonal imbalances, inflammatory processes and behavioural changes (figure 1, Panel B).

Two reviews investigated HbA1c among people who smoke and people who used to smoke.^32 46^ As expected, smoking was found to be associated with poor cardiometabolic outcomes including HbA1c, and smoking acted as a risk factor independent of other factors. Compared with current smoking, being a person who used to smoke was related to better cardiometabolic factors, with two studies reporting a HbA1c mean difference of −0.1 between people who smoke and people who used to smoke.^46^ However, both studies reported that people who have never smoked had better HbA1c than people who smoke and people who used to smoke.^32 46^ Mechanisms suggested included pancreatic damage, increased inflammation and oxidative stress (figure 1, Panel C).

Only one review investigated observational studies of diet quality indices and HbA1c.^48^ The review found that while most dietary quality indices were negatively correlated with HbA1c levels, the strongest correlations were with the US Department of Agriculture Healthy Eating Index (HEI) (correlation coefficient: −0.35) and the Mediterranean Diet Score (MDS) (correlation coefficient: −0.28).

**Figure 2:**
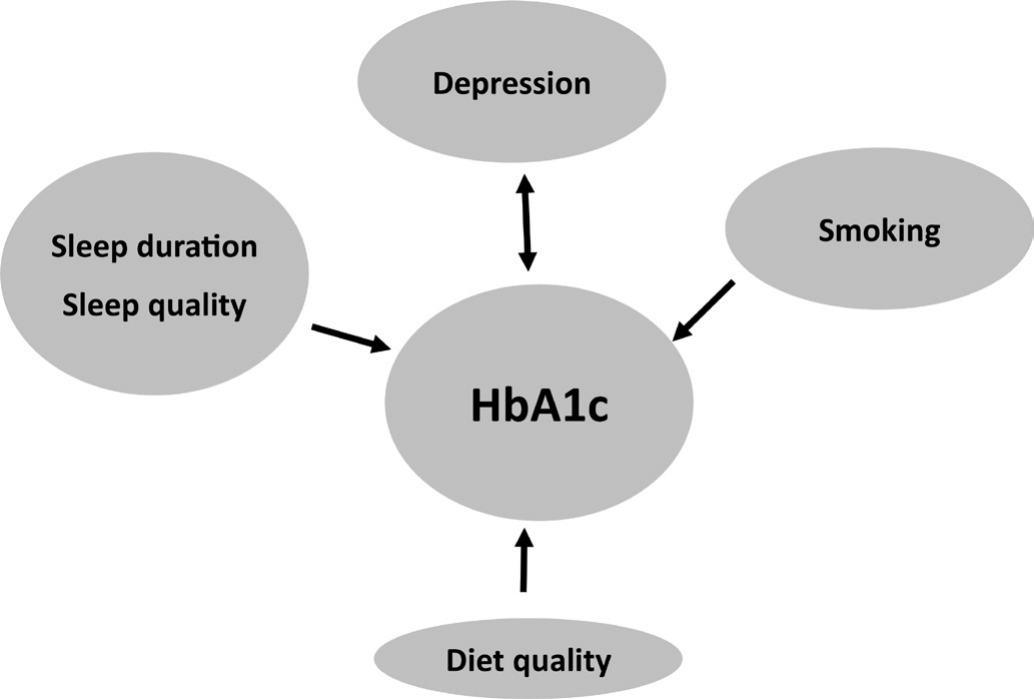
Summary of relationships between health risk behaviours, mental health and HbA1c as found in this review. HbA1c, glycated haemoglobin.

The AMSTAR-2 risk of bias assessment tool^41^ did not identify any significant issues with the reviewed papers and Kohen’s Kappa of the scores between the two reviewers was 0.81 (online supplemental appendix A, table A.1). Heterogeneity reported in the examined studies varied from a high of 100%^44^ to a low of 38%.^6^ However, for any given factor such as sleep or depression, there were a mix of heterogeneity values with, for instance, the sleep-HbA1c relationship having heterogeneity from 54% to 100% (online supplemental appendix B, table B.1). Figure 2 summarises the relationships described above between the various HRBs, depression and HbA1c.

## Discussion

In this umbrella review, one of the first of its kind, we report on the association between HbA1c and six key health risk factors while focussing specifically on observational studies in real-world conditions. Our meta-review found relationships in the expected direction with greater HbA1c or HbA1c variability being associated with depression, abnormal or poor sleep patterns, poor quality diet and smoking. The relationship between HbA1c and depression was bidirectional. Concerningly, there were no systematic reviews of observational studies that focused on examining alcohol intake, exercise or anxiety. While a number of umbrella reviews have explored HbA1c as an outcome, often as an indicator of diabetes, their focus has generally been clinical,^50^ often unpacking the effects of specific clinical or health service interventions.^50 51^ When non-clinical indicators are investigated, the study design is often an RCT, with us finding only eight studies that were observational.

For some of the investigated indicators/interventions, our observational study-related findings generally agree with existing literature that includes RCTs. Thus, one such umbrella review investigated the effect of multiple interventions such as diet and exercise to preventative education on HbA1c.^52^ Another umbrella review specifically underscored the relevance of special diets in reducing HbA1c.^53^ The study found that the lifestyle interventions had a positive impact on HbA1c levels among other outcomes. Various systematic reviews of RCTs support our findings on worse HbA1c with smoking.^54^ Similarly, systematic reviews of RCTs show that interventions that reduce symptoms of depression also lead to improvements in HbA1c.^55 56^ While our analyses generally agree with results from existing RCTs, this does not necessarily imply that results of such evidence syntheses of observational studies in all disciplines and domains will agree with conclusions from RCTs. Thus, for instance, a systematic review of analyses from the large longitudinal Nurses’ Health Study in the USA found poor concordance between the results of observational analyses and those from RCTs, with agreement observed in only about 25% of cases.^57^ Variations in triangulated knowledge from different sources of knowledge, either complementary or contradictory, offer a more comprehensive understanding of the phenomenon at hand, including the areas of uncertainty.

Substantively then, our study supports and summarises an existing body of research indicating that poor lifestyle choices are associated with higher HbA1c levels. Conversely, the results support the role of better sleep, special diets, physical activity, not smoking and better mental health in the maintenance of normal and steady HbA1c levels. This, in turn, can help support accumulating evidence in the literature on allostatic load, of which HbA1c is one of the many metrics.^30^ Since existing policies already tend to encourage some of the lifestyle choices that support better HbA1c levels, our findings should help support further policies in this direction and bolster the research base for existing policies.^58^

The findings from this study also have other research and policy implications. First, it underscores the paucity of research or syntheses of research that use an observational study design in the investigation of relationships between HRBs, mental health and HbA1c with observational studies on key HRBs such as physical activity, diet and alcohol intake or mental health outcomes such as anxiety not having been subject to any synthesis thus far. This paucity may underscore a blind adherence to the concept of a hierarchy of evidence and the lack of consilience in assessing and integrating scientific evidence that may be perceived as less rigorous (eg, observational studies).^22^ Syntheses of observational studies should be a key area of future work. For example, syntheses could focus on longitudinal designs, which allow for the studying of temporal precedence and appropriate time-zero assignments.^24 29^ As underscored earlier, while RCTs can provide valuable insights on the efficacy of, for instance, a specific diet in reducing HbA1c, observational studies can provide evidence on the effectiveness of the diet within a complex real-world environment that may include the intake of other foods and HRBs in reducing HbA1c in the broader population.^12 22^ Various forms of evidence, including RCTs, observational studies, cultural knowledge, expert knowledge and experiential knowledge, can then inform further research and policymaking.^1922^,^24^

This review covers a comprehensive and unique set of search terms, being one of the first reviews of its kind. Based on our findings, there is scope for further analysis and expansion, such as a search including the Medical Subject Headings (MeSH) term HbA1c (plus the systematic review filter, then screening for reviews which discuss HRBs reducing risk of omitting relevant reviews. Another possible scope for expansion is the use of the (tw) term in search or the including of (MeSH), which when used with caution, can support a more comprehensive review.^59^ While our study uses a title and abstract search following some other umbrella reviews,^60 61^ it may also be beneficial to implement the initial search on full text, although the addition of MeSH terms did not reduce sensitivity in the majority (59%) of studies.^62^ Spelling variations such as ‘haemoglobin’ may also be incorporated. Also, including studies not written in English also provides an opportunity for expansion. While a meta-analysis could not be implemented in the current review because of the heterogeneity of the reviewed studies, it may be possible to perhaps select a smaller set of relatively homogenous studies to implement a meta-analysis on a study set from an expanded group of search terms. This may especially be feasible if one specific lifestyle or health issue (such as depression) and its relationship with HbA1c is being examined. Future research can thus expand on the current study to examine both in depth and in breadth the current state of knowledge on the drivers and correlates of healthy HbA1c levels. While there is potential for limitations of confounding, reverse causation, design-related and other potential biases, the results from this umbrella review largely agree with what has been found in other reviews that focus on RCTs and identify gaps and under-researched areas that require further investigation.^26^ This review provides convergent and consilient evidence on the drivers and correlates of HbA1c.

## Supplementary material

10.1136/bmjopen-2024-092657 online supplemental file 1

## Data Availability

No data are available.
