# Supplementary material for: Health risk behaviours, mental health and HbA1c: an overview of reviews of observational studies
Source: BMJ Open. 2025 Nov 16;15(11):e092657. doi: 10.1136/bmjopen-2024-092657 (PMC12625968; doi:10.1136/bmjopen-2024-092657)
Supplement: online supplemental file 1 [file bmjopen-15-11-s001.docx]

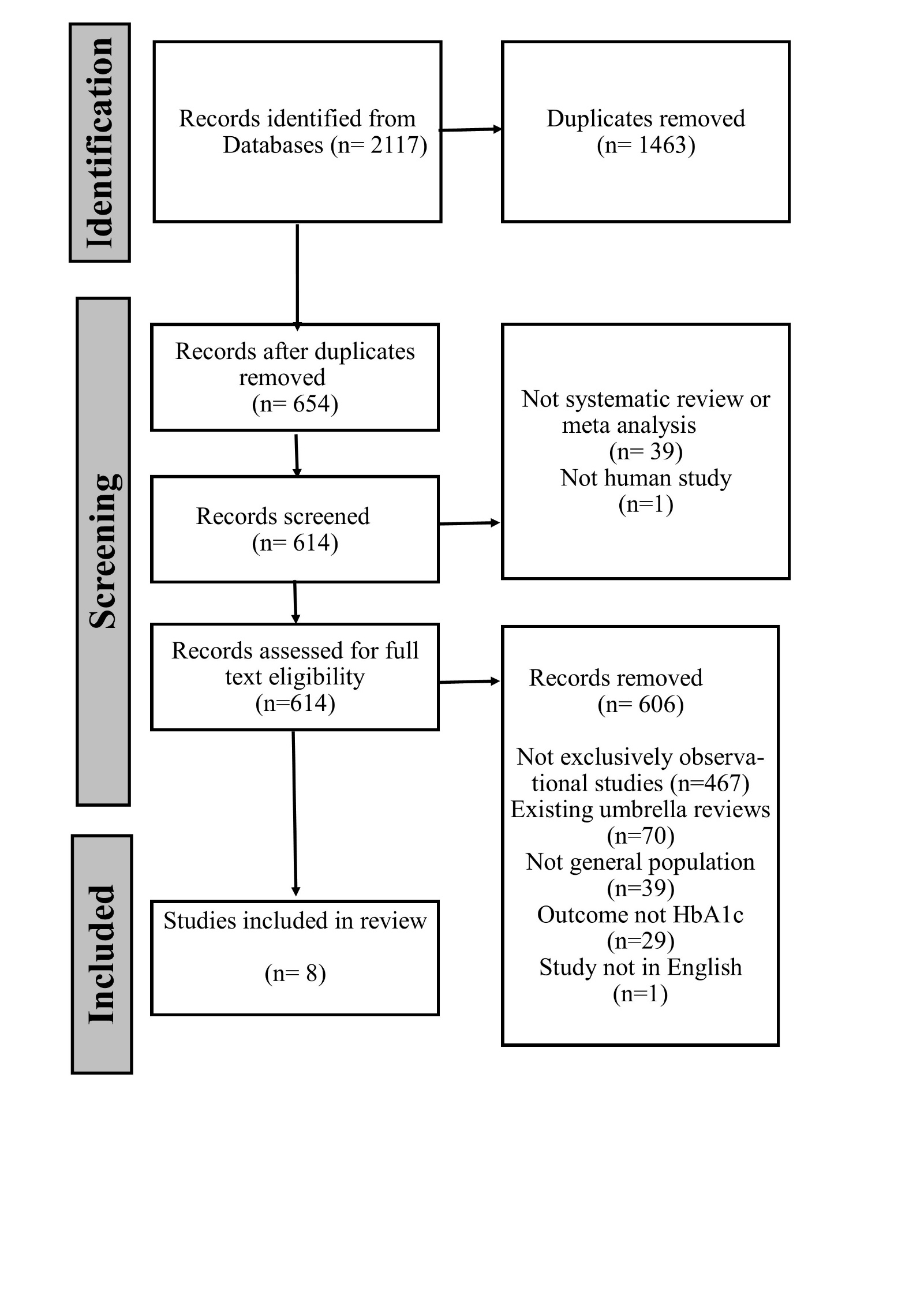
**Figure S.1** **Figure S.1. Flow-chart of articles excluded in the umbrella review.**

**Appendix A:**

**SCOPUS ADVANCED SEARCH STRING**

(TITLE-ABS("Sleep disorder*" OR "Sleep wake disorder*" OR "Sleep deprivation" OR "Sleep arousal" OR "Insomnia" OR "Obstructive sleep apnea" OR "Anxiety" OR "Mood disorder" OR "Stress" OR "Psychological distress" OR "depressive disorder" OR depress* OR "Depressive disorder" OR "Depressive symptom" OR "Dysthymic disorder" OR "alcohol" OR "smoking" OR "diet" OR "Physical activity" OR "sedentarism"))

AND

(TITLE-ABS(HbA1c OR "Hemoglobin A1C" OR "Glycated Hemoglobin"))

AND

(SRCTITLE(systematic) OR DOCTYPE(re))

**WEB OF SCIENCE ADVANCED SEARCH STRING**

TS=("Sleep disorder*" OR "Sleep wake disorder*" OR "Sleep deprivation" OR "Sleep arousal" OR Insomnia OR "Obstructive sleep apnea" OR Anxiety OR "Mood disorder" OR Stress OR "Psychological distress" OR depress* OR "Depressive disorder" OR "Depressive symptom" OR "Dysthymic disorder" OR alcohol OR smoking OR diet OR "Physical activity" OR sedentarism)

AND

TS=(HbA1c OR "Hemoglobin A1C" OR "Glycated Hemoglobin")

AND

DT=(“Review” OR “Meta-Analysis”) AND TS=(systematic)

**PSYCHINFO ADVANCED SEARCH STRING**

((Sleep disorder* or Sleep wake disorder* or Sleep deprivation or Sleep arousal or Insomnia or Obstructive sleep apnea or Anxiety or Mood disorder or Stress or Psychological distress or depressive disorder or depress* or Depressive disorder or Depressive symptom or Dysthymic disorder or alcohol or smoking or diet or Physical activity or sedentarism).ti,ab.)

AND

(HbA1c or Hemoglobin A1C or Glycated Hemoglobin).ti,ab.

AND

(systematic review or literature review or meta-analysis).mp.

**EPISTEMONIKOS SEARCH STRING**

(title:(("Sleep disorder*" OR "Sleep wake disorder*" OR "Sleep deprivation" OR "Sleep arousal" OR "Insomnia" OR "Obstructive sleep apnea" OR Anxiety OR "Mood disorder" OR Stress OR "Psychological distress" OR "depressive disorder" OR depress* OR "Depressive disorder" OR "Depressive symptom" OR "Dysthymic disorder" OR alcohol OR smoking OR diet OR "Physical activity" OR sedentarism) AND (HbA1c OR "Hemoglobin A1C" OR "Glycated Hemoglobin")) OR abstract:(("Sleep disorder*" OR "Sleep wake disorder*" OR "Sleep deprivation" OR "Sleep arousal" OR "Insomnia" OR "Obstructive sleep apnea" OR Anxiety OR "Mood disorder" OR Stress OR "Psychological distress" OR "depressive disorder" OR depress* OR "Depressive disorder" OR "Depressive symptom" OR "Dysthymic disorder" OR alcohol OR smoking OR diet OR "Physical activity" OR sedentarism) AND (HbA1c OR "Hemoglobin A1C" OR "Glycated Hemoglobin")))

**DATABASES IN WEB OF SCIENCE SEARCH**

Web of Science Core Collection

Current Contents Connect

Chinse Science Citations Database

KCI-Korean Journal Database

MEDLINE

SciELO Citations Index

**TERMS INCLUDED IN THE sb FILTER IN PUBMED**

As of May 2025, the terms included via the Systematic[sb] filter in PubMed, are as follows:

(((systematic review[ti] OR systematic literature review[ti] OR systematic scoping review[ti] OR systematic narrative review[ti] OR systematic qualitative review[ti] OR systematic evidence review[ti] OR systematic quantitative review[ti] OR systematic meta-review[ti] OR systematic critical review[ti] OR systematic mixed studies review[ti] OR systematic mapping review[ti] OR systematic cochrane review[ti] OR systematic search and review[ti] OR systematic integrative review[ti]) NOT comment[pt] NOT (protocol[ti] OR protocols[ti])) NOT MEDLINE [subset]) OR (Cochrane Database Syst Rev[ta] AND review[pt]) OR systematic review[pt]

**A.1 (Table)**

AMSTAR scoring table.

| **Question number>**  **Author** | **Q1** | **Q2** | **Q3** | **Q4** | **Q5** | **Q6** | **Q7** | **Q8** | **Q9** | **Q10** | **Q11** | **Q12** | **Q13** | **Q14** | **Q15** | **Q16** |
| --- | --- | --- | --- | --- | --- | --- | --- | --- | --- | --- | --- | --- | --- | --- | --- | --- |
| **Beran et al (2021)** | √ | √ | √ | √ | √ | √ | √ | X | √ | √ | √ | √ | √ | √ | X | √ |
| **Genis-Mendoza et al (2022)** | √ | √ | √ | √ | √ | √ | X | √ | √ | √ | √ | √ | √ | √ | √ | √ |
| **Kar et al (2016)** | √ | √ | √ | √ | √ | √ | √ | √ | √ | √ | √ | √ | √ | √ | √ | √ |
| **Koopman et al (2020)** | √ | √ | √ | √ | X | √ | √ | √ | √ | √ | √ | √ | √ | √ | √ | √ |
| **Lee et al (2017)** | √ | √ | √ | √ | √ | √ | √ | √ | √ | √ | √ | √ | √ | √ | √ | √ |
| **Sepandi et al (2022)** | √ | √ | √ | √ | X | √ | √ | √ | √ | √ | √ | √ | √ | X | √ | √ |
| **Soulimani et al (2014)** | √ | √ | √ | √ | √ | √ | √ | √ | √ | √ | √ | √ | √ | √ | √ | √ |
| **Zhu et al (2017)** | √ | √ | X | √ | √ | √ | √ | X | √ | √ | √ | √ | X | X | √ | √ |

**List of Questions**

| Q1. Did the research questions and inclusion criteria for the review include the components of PICO/PECO? |
| --- |
| Q2. Did the report of the review contain an explicit statement that the review methods were established prior to the conduct of the review and did the report justify any significant deviations from the protocol? |
| Q3. Did the review authors explain their selection of the study designs for inclusion in the review? |
| Q4. Did the review authors use a comprehensive literature search strategy? |
| Q5. Did the review authors perform study selection in duplicate? |
| Q6. Did the review authors perform data extraction in duplicate? |
| Q7. Did the review authors provide a list of excluded studies and justify the exclusions? |
| Q8. Did the review authors describe the included studies in adequate detail? |
| Q9. Did the review authors use a satisfactory technique for assessing the risk of bias (RoB) in individual studies that were included in the review? |
| Q10. Did the review authors report on the sources of funding for the studies included in the review? |
| Q11. If meta-analysis was performed did the review authors use appropriate methods for statistical combination of results? |
| Q12. If meta-analysis was performed, did the review authors assess the potential impact of RoB in individual studies on the results of the meta-analysis or other evidence synthesis? |
| Q13. Did the review authors account for RoB in individual studies when interpreting/ discussing the results of the review? |
| Q14. Did the review authors provide a satisfactory explanation for, and discussion of, any heterogeneity observed in the results of the review? |
| Q15. If they performed quantitative synthesis did the review authors carry out an adequate investigation of publication bias (small study bias) and discuss its likely impact on the results of the review? |
| Q16. Did the review authors report any potential sources of conflict of interest, including any funding they received for conducting the review? |

**Appendix B:**

**B.1 (Table)**

Heterogeneity in examined studies/relationships.

| **Author (Year): Exposure** | | **I squared** | **Q** | **p** | **Eggers test p** |
| --- | --- | --- | --- | --- | --- |
| Genis-Mendoza et al (2022): Depressed versus not depressed | 85% | | NA | NA | NA |
| Genis-Mendoza et al (2022): Depression less than 10 years | 66% | | NA | 0.001 | NA |
| Genis-Mendoza et al (2022): Depression more than 10 years | 69.33 | | NA | 0.14 | NA |
| Beran et al (2021) Depressive symptoms | 38% | | 7.94 | 0.16 | NA |
| Koopman et al (2020): Sleep quality (Symptoms of insomnia - dichotomized HbA1c levels) | 100% | | NA | 0.00001 | NA |
| Lee et al (2016): Sleep schedule (Sleep duration - short) | 54% | | 12.99 | 0.04 | 0.06 |
| Lee et al (2016): Sleep schedule (Sleep duration - long) | 55% | | 13.45 | 0.04 | 0.62 |
| Lee et al (2016): Sleep quality (Sleep quality - overall poor sleep) | 65% | | 22.84 | 0.004 | 0.32 |
| Kar et al (2016) (Quitters versus smokers) | 93% | | 44.07 | 0.00001 | NA |
| Kar et al (2016) (Non-smokers versus smokers) | 98% | | 477.82 | 0.00001 | NA |
| Soulimane et al (2014) (Never smokers versus current smokers) | 55% | | NA | NA | NA |
| Soulimane et al (2014) (Quitters versus smokers) | 93% | | NA | NA | NA |
