# Supplementary material for: Health risk behaviours, mental health and HbA1c: an overview of reviews of observational studies
Source: BMJ Open. 2025 Nov 16;15(11):e092657. doi: 10.1136/bmjopen-2024-092657 (PMC12625968; doi:10.1136/bmjopen-2024-092657)
Supplement: Supplementary file 2 [file bmjopen-2024-092657.reviewer_comments.pdf]

PEER REVIEW HISTORY

BMJ Open publishes all reviews undertaken for accepted manuscripts. Reviewers are asked to complete a checklist review form and are provided with free text boxes to elaborate on their assessment. These free text comments are reproduced below.

ARTICLE DETAILS

Title (Provisional)

Health Risk Behaviours, Mental Health and HbA1c: An overview of reviews of observational studies

Authors

Mazumdar, Soumya; Almeda, Nerea; Bagheri, Nasser; Daniel, Mark; Tabatabaei Jafari, Hossein; Leigh, Gweneth; Diaz Milanes, Diego; Salvador-Carulla, Luis

---

VERSION 1 - REVIEW

**Reviewer**	**1**
**Name**	**Dorans, Kirsten S**
**Affiliation**	**Tulane University**
**Date**	**06-Oct-2024**
**COI**	

For EDITOR ONLY: Though this is an important topic, there are a number of major concerns that I have and very substantial revisions recommended; nearly bordering on rejection. The authors leave out core components of limitations of observational studies and barely mention how RCTs can be randomized at cluster level. Biggest concern though is that the methods are a bit vague/general, search strategy may not be sufficient (this is not my expertise so recommend someone with expertise in systematic reviews review), and it is unclear how certain findings were chosen to be highlighted in results (noted in comments to author below).

Additionally, there is limited about amount of data presented in supplemental material given how many articles were reviewed and figure does not include units for A1c, which leads to concern about attention to detail and whether some reviews reported A1c in % and others in mmol/L, which is important when reporting magnitude of associations.

I do not think authors provided a relevant checklist.

AUTHOR REVIEWS:

This is an important research topic and worthwhile to evaluate observational studies in this topic area. However, there are some major concerns primarily around (a) search terms/approach used may not be sufficient (recommend reference librarian review for completeness); (b) key limitations of observational studies and, as a result, findings from this article not mentioned (e.g., confounding); (c) unclear how some results obtained (e.g., 2% of articles being observational - was this from all of the articles across all of the reviews included?)

Comments:

1) Abstract - if only observational studies were included, what is meant by "Only two percent of overall studies were observational studies" in the abstract?

2) Intro "While RCTs randomize across individual characteristics" - as noted below there are also cluster RCTs. Recommend revising this sentence to state something more like "Individually randomized RCTs randomize at individual level, which ..." and also describing cluster RCTs here.

- What exactly is meant by 'contextual environments'?

- For the example on walkable neighborhoods, couldn't an RCT of a physical activity intervention be studied across people in both types of neighborhoods and look at effects of intervention stratified by neighborhood type?

- Recommend to describe how RCTs have variability in how tightly controlled they are - for instance effectiveness-focused vs efficacy-focused RCTs (effectiveness ones trying to be more like real-world)

3) Regarding Hawthorne effect, recommend adding citation for first sentence where discuss it.

4). "inform evidence informed decisions" recommend instead saying "inform evidence-based decisions"

5) The major limitations of confounding and bias from observational studies should be addressed in the introduction.

6) Why was the search only started in 2013?

7) For the search terms, was "hemoglobin A1c" used? "glycated haemoglobin" used? Were any MESH terms used? Why were search terms restricted to title/abstract (for systematic reviews, it's likely that exposure or A1c may not be included in title/abstract but still reported in results)?

8) In results, "recommend that increased risk" not be used because it may make it sound more causal and (a) many studies are cross-sectional and (b) these are observational studies. Instead could say associated with an X% higher risk of ...

9) For methodology, how was it determined what measures of association would be highlighted in results - for example the 18% higher risk of depression associated with 1 point higher A1c -- was this the only review that provided this type of association?

10) For A1c units, did all report A1c as % vs mmol/l (SI units)? Which unit for A1c is being used in Figure 1?

11) Does this refer to CCA metric of less than 2% noted in results? Can you provide more details beyond brief sentence/reference of what a CCA metric is?

12) In discussion - can you provide a reference for this sentence: Thus, for instance a review of analyses from the large longitudinal Nurses' Health Study in

the USA found poor agreement between results from the study and RCT

13) Authors note all data included in paper but was there any additional data extracted across the articles that was used to inform results/discussion?

**Reviewer**	**2**
**Name**	**Ellis, Ursula**
**Affiliation**	**The University of British Columbia, Woodward Library**
**Date**	**10-May-2025**
**COI**	

Thank you for the opportunity to review your work. My comments focus on the search methods and reporting. The umbrella review appears to be novel, with an extensive rationale provided for why an overview of reviews of observational studies on this topic is needed. Registration of the protocol in PROSPERO is another strength.

I do have some concerns with the review:

1. I think that the search terms are not comprehensive enough to achieve the review's goals. Only the PubMed search is reported, so I can't assess the strategy used in other databases. In the PubMed search, most concepts are searched only in title and abstract fields; a few MeSH

terms are included, but concerningly, not the MeSH term Glycated Hemoglobin. One of the free text terms reported for HbA1c appears to be a typo: "Hemoglobine A1c"[tiab] returns only 4 results in PubMed. The variant spelling haemoglobin is not included.

Additionally, the terms for the 6 key health risk factors are very restricted. For example, the term smoking is searched in title and abstract, but not tobacco or nicotine; diet, but not dietary or diets or other diet-related terms (which could miss key reviews on for instance, sugar intake); physical activity and sedentarism, but not exercise or sedentary time/behaviour. There are terms for anxiety, depression, mood disorder, stress and distress, but the term "mental health" is not included. The lack of MeSH terms for most of the health risk behaviours may have led to missing relevant reviews, since some articles lack abstracts. There could have been more use of the * after terms to find various word endings, such as after mood disorder* or depressive symptom*.

An additional valuable field that could have been searched for free text is author-provided keywords. The text word [tw] field code in PubMed searches for matches in title, abstract, author-provided keywords, and MeSH fields. Please note though that it is best practice to also search MeSH terms in addition to terms in [tw], as using the MeSH will expand your search to include narrower, related MeSH terms. As an example, a search for Diet[MeSH] will include all the terms listed at the bottom of this page - https://www.ncbi.nlm.nih.gov/mesh/68004032 - so could find a review on intermittent fasting's impact on HbA1c where the term "diet" might not be used in the title or abstract.

A very comprehensive search might include a search only for HbA1c (including the MeSH term for it) plus the systematic review filter, then screening for reviews which discuss health risk behaviours. The screening burden would certainly be higher using this approach, but it runs less risk of omitting relevant reviews.

2. Searches could be reported more clearly.

Item 7 in the PRIOR checklist says "Present the full search strategies for all databases, registers and websites, such that they could be reproduced. Describe any search filters and limits applied." Please add to the supplement the exact search terms used in Scopus, Web of Science, and PsycINFO-PsycArticles. Further, for Web of Science, please list which databases were included in your search (such as Science Citation Index, Social Sciences Citation Index). Databases in Web of Science can vary a lot from one institution to another. Also, please indicate which platform PsycInfo/PsycArticles were searched through - EBSCO? Ovid?

For greater transparency, I recommend reporting the terms used in the Systematic[sb] filter in PubMed, as this filter may change over time or be removed. The terms included as of May 9, 2025 are:

(((systematic review[ti] OR systematic literature review[ti] OR systematic scoping review[ti] OR systematic narrative review[ti] OR systematic qualitative review[ti] OR systematic evidence review[ti] OR systematic quantitative review[ti] OR systematic meta-review[ti] OR systematic critical review[ti] OR systematic mixed studies review[ti] OR systematic mapping review[ti] OR systematic cochrane review[ti] OR systematic

search and review[ti] OR systematic integrative review[ti]) NOT comment[pt] NOT (protocol[ti] OR protocols[ti])) NOT MEDLINE [subset]) OR (Cochrane Database Syst Rev[ta] AND review[pt]) OR systematic review[pt]

(https://www.nlm.nih.gov/bsd/pubmed_subsets/sysreviews_strategy.html)

Note that the filter does not effectively find scoping reviews, rapid reviews, or some meta-analyses (those which may not mention the term systematic in the title or be tagged with the publication type). While you were only specifically looking for systematic reviews or meta-analyses, I noted that one integrative review was included in your umbrella review.

3. Search sources and dates

The review would be strengthened with an updated search to include any studies published or added to databases since Sept 2023. I strongly recommend updating the search, and also expanding the search to include Epistemonikos, a specialty database of systematic reviews: https://www.epistemonikos.org/ I would advocate using a simple search in Epistemonikos, such as just HbA1c and its synonyms, and screening the results.

Also, I wonder if you could explain why the search was limited to 2013-present. Given that there seem to be few reviews covering only observational studies, why not look back farther?

I have some more minor comments for your consideration:

4. You state that the search was performed by 2 researchers. This isn't necessary, unlike other review steps like screening, data extraction and quality assessment that benefit from two people's involvement in order to limit bias. While two people or the whole team might have been involved in developing the search, it's sufficient for one person to run the final search and export results to the tool you are using for screening.

5. Could you please report how deduplication was done? Eg Covidence, Endnote, some other process? This is one of the items noted in the PRISMA-S extension, which may be of interest: https://doi.org/10.1186/s13643-020-01542-z

6. While the search was not limited to English, I noticed in the PRISMA flow diagram 1 non-English study was excluded. I suggest stating the restriction to English in your eligibility criteria and in the limitations. Also, Table 1 appears to be missing from the manuscript.

7. On page 5 you mention the "Cochrane Pyramid" - this term wasn't familiar to me, it might be clearer to call it the EBM pyramid? I know what you're referring to, but I'm unclear that it has its origin with Cochrane or is generally known by that name.

8. Perhaps I missed it but I did not find a completed PRIOR checklist in the supplementary materials.

VERSION 1 - AUTHOR RESPONSE

Reviewer 1

Comment: This is an important research topic and worthwhile to evaluate observational studies in this topic area. However, there are some major concerns primarily around (a) search terms/approach used may not be sufficient (recommend reference librarian review for completeness); (b) key limitations of observational studies and, as a result, findings from this article not mentioned (e.g., confounding); (c) unclear how some results obtained (e.g., 2% of articles being observational - was this from all of the articles across all of the reviews included?)

Response: Thank you. We would like to thank the reviewer for the supportive comments and while the comments from the reviewer are addressed in later sections, we have summarised some the changes below.

A) Regarding search terms

- We prioritised a focused and conceptually coherent search strategy to ensure feasibility and alignment with our predefined framework.
- We used eVITAL, a published taxonomy to guide term selection.
- We included MeSH terms where appropriate.
- We avoided using [tw] for MeSH terms due to concerns about reduced recall, citing Salvador-Oliván et al. (2019).
- We acknowledged that broader terms and more wildcard use (e.g., diet*) could have improved recall.
- We added a limitation in the discussion noting the potential for missed studies due to the search scope.

The key issue is that being an umbrella review our aim was to synthesise existing systematic reviews rather than conduct an exhaustive primary-level search. Therefore, we prioritised a focused and conceptually coherent search strategy to ensure feasibility, manageability, and alignment with our predefined framework. Overly broad search terms would have significantly increased the volume of irrelevant results, potentially diluting the specificity and clarity of our synthesis. It is also important to note that there are fundamental differences between a systematic review an umbrella review such as this. An umbrella review follows a simpler search strategy than a systematic review. Thus according to experts on this topic (1) "Search strategies for reviews of reviews will be simpler than those for systematic reviews of single studies as the included reviews will already have undertaken these searches (pg 86)….". See response to comment 9 below. Also, a librarian was consulted for the search, and we have now included this statement in the "study design" section "The process was supported by a research librarian."

(b) Limitations of Observational Studies (e.g., Confounding)

Our Response: We added a sentence in the introduction explicitly noting:

“Indeed, while some RCT designs such as cluster randomised designs can incorporate context to an extent, observational studies that account for confounding and bias, are better positioned to account for the Hawthorne effect, and the great multitude of heterogenous contexts and populations, across which it may be difficult or unethical to define clusters and randomise.”

(c) Clarification of “2% of articles being observational”

Our Response: We removed the sentence from the abstract and elsewhere.

Comment:) Abstract - if only observational studies were included, what is meant by "Only two percent of overall studies were observational studies" in the abstract?

Response: Thank you. This was an error. We have removed this sentence

Comment: 2) Intro "While RCTs randomise across individual characteristics" - as noted below there are also cluster RCTs. Recommend revising this sentence to state something more like "Individually randomised RCTs randomise at individual level, which ..." and also describing cluster RCTs here.
- What exactly is meant by 'contextual environments'?
- For the example on walkable neighborhoods, couldn't an RCT of a physical activity intervention be studied across people in both types of neighborhoods and look at effects of intervention stratified by neighborhood type?
- Recommend to describe how RCTs have variability in how tightly controlled they are - for instance effectiveness-focused vs efficacy-focused RCTs (effectiveness ones trying to be more like real-world)

Response: Thank you very much for these insightful comments that have helped us improve the overall writing quality of this paper. We have incorporated the "While RCTs randomise across individual characteristics" comment as follows:

Introduction Section->Second Paragraph-> Third Sentence now reads as follows:

“While individually randomised RCTs randomise across individual levels, and may investigate subgroup effects through stratification, it is difficult to randomise across contextual environments without recourse to complex designs such as cluster randomised trials.”

We now clarify contextual factors in the paper, first paragraph->Last Sentence as follows:

“In this regard contextual environments encompass the broader social and environmental conditions that shape health and illness, and are essential to understanding the complex, multifactorial nature of health outcomes [5].”

With regard to the reviewers comments on RCT designs, to accommodate the reviewers concerns we have included the following sentences at the end of Introduction->Second Paragraph

“While it is true that RCTs—particularly those designed with an effectiveness focus—can attempt to emulate real-world conditions and may stratify analyses by contextual variables such as neighbourhood type, such designs still face practical and ethical constraints in randomising across diverse environmental settings. Moreover, the degree of control in RCTs varies along a spectrum from tightly controlled efficacy trials to more pragmatic effectiveness trials, with the latter aiming to enhance external validity but still often falling short of capturing the full complexity of real-world, heterogeneous environments. Observational studies, by contrast, are inherently suited to capturing these complexities. They operate in fully naturalistic settings, allowing for the study of diverse populations and contextual interactions without the constraints of intervention protocols or randomisation. Unlike pragmatic trials, which still require structured intervention delivery and monitoring, observational studies can investigate exposures that are unethical or impractical to randomise. They also allow for larger sample sizes, lesser costs, and longer follow-up durations, making them particularly valuable for studying chronic conditions and rare outcomes. Furthermore, modern analytical techniques such as propensity score matching and Mendelian Randomisation enhance the causal inference capabilities of observational designs, offering complementary insights to those derived from RCTs (2, 3)”

Comment: 3) Regarding Hawthorne effect, recommend adding citation for first sentence where discuss it.

Response: Thank you very much. We have made this change.

“In addition, when the intervention is something such as lifestyle change or exercise, RCTs are subject to the Hawthorne effect” with the following reference: Berkhout C, Berbra O, Favre J, et al. Defining and evaluating the Hawthorne effect in primary care, a systematic review and meta-analysis. Frontiers in Medicine 2022;9:1033486.

Comment: 4). "inform evidence informed decisions" recommend instead saying "inform evidence-based decisions"

Response: Thank you very much for allowing us the opportunity for clarification. Evidence informed in this context implies moving away from RCT based EBM approaches. To better clarify this, we have included the following text to the aforementioned paragraph:

“Instead, many forms of knowledge sources such as observational studies, contextual evidence, cultural norms, expert insights, and consumer experiences, in addition to RCTs could support policy development and implementation or support evidence informed decisions[12 13]. Evidence-informed decision-making reflects a shift away from rigid hierarchies of evidence towards a more inclusive approach that values diverse types of knowledge. According to the World Health Organisation, this approach emphasises identifying, appraising, and mobilizing the best available evidence—including qualitative and contextual data—to inform policies and programs[14].”

Comment: 5) The major limitations of confounding and bias from observational studies should be addressed in the introduction.

Response: Thank you. First Section->Third Paragraph->Fourth Sentence now reads as follows:

“Indeed, while some RCT designs such as cluster randomised and pragmatic designs can incorporate context to an extent, observational studies that account for confounding and bias, are better positioned to account for the Hawthorne effect, and the great multitude of heterogenous contexts and populations, across which it may be difficult or unethical to define clusters and randomise.”

Comment: 6) Why was the search only started in 2013?

Response: Thank you. At the time of this review, 2013 corresponded to 10 years of literature from 2023 (which was our cut off date). This was to access the latest literature on the topic. The first paragraph of Study Design and Methods->Information source and search strategy now reads as follows:

“The search was implemented on Pubmed, Scopus, Web of Science and PsycINFO-PsychArticles from January 2013 to 31 May 2025. This corresponds to approximately a 12-year time window, allowing us to assess relatively contemporary literature on the topic. Additionally, the reference lists of included articles were reviewed manually for any additional articles.”

Comment: 7) For the search terms, was "hemoglobin A1c" used? "glycated haemoglobin" used?

Response: Thank you. Both terms were used in the search. The last two terms in the search string reads as follows:

“Hemoglobin A1C"[Title/Abstract] OR "Glycated Hemoglobin"[Title/Abstract])).”

The spelling variation of “haemoglobin” was not incorporated. Nevertheless, our search yielded results with this variation of spelling, an example is provided below:

Muñoz-Pardeza, J., López-Gil, J. F., Huerta-Uribe, N., Hormazábal-Aguayo, I., Izquierdo, M., & García-Hermoso, A. (2024). Nonpharmacological interventions on **glycated haemoglobin** in youth with type 1 diabetes: a Bayesian network meta-analysis. *Cardiovascular Diabetology*, *23*(1), 230.

While ideally both the American and British variants of the search term could be used, the American term appears to be used more substantially often in the literature. Thus for example while a PubMed search in the last ten years of “hemoglobin” in the literature gives us 8,304 results, searching for “haemoglobin” produces only 1,549 results. Nevertheless, to acknowledge this as a limitation we have included the following sentence in the limitations section:

“Spelling variations such as “haemoglobin” may also be incorporated”

Comment: Were any MESH terms used? Why were search terms restricted to title/abstract (for systematic reviews, it's likely that exposure or A1c may not be included in title/abstract but still reported in results)?

Response: Thank you for the opportunity to clarify our search approach. A number of terms in our search are MeSH Terms as follows:

Sleep Disorders

Sleep Wake Disorders
Sleep Deprivation
Insomnia
Obstructive Sleep Apnea
Anxiety
Mood Disorders
Stress, Psychological
Psychological Distress
Depressive Disorder
Dysthymic Disorder
Smoking
Diet

Also, in an umbrella review of systematic reviews (unlike a systematic review) we expect the key findings of the specific systematic review to be highlighted in the title and/or the abstract. As such it is not uncommon for umbrella reviews to limit the search to Title and Abstract. For example, see:

Favril, L., Rich, J. D., Hard, J., & Fazel, S. (2024). Mental and physical health morbidity among people in prisons: an umbrella review. ***The lancet public health***, *9*(4), e250-e260.

Nevertheless, to acknowledge this fact we have modified the limitations section – Discussion->Last Paragraph->First Sentence as follows:

"While this review covers a comprehensive and unique set of search terms, being one of the first reviews of its kind, there is scope for further analysis and expansion, such as the expansion of the initial search from beyond title and abstract."

Comment: 8) In results, "recommend that increased risk" not be used because it may make it sound more causal and (a) many studies are cross-sectional and (b) these are observational studies. Instead, could say associated with an X% higher risk of ...

Response: Thank you very much for this feedback. We have made the change and the sentence now reads as follows:

"A one-point increase in HbA1c was associated with an 18% higher risk of depression, while a one-unit increase in depressive symptoms was associated with a 0.07 unit increase in HbA1c (Figure 1-Panel A)"

Comment: 9) For methodology, how was it determined what measures of association would be highlighted in results - for example the 18% higher risk of depression associated with 1 point higher A1c -- was this the only review that provided this type of association?

Response: Thank you very much for allowing us to clarify further. Please note that the reviewed research papers are meta-analyses and systematic reviews, since this is an umbrella review. As such, summary measures in the reviews are reported. We have tried to report as much as possible from the reviews examined. Two studies examined the relationship between HbA1c and depression. One by Genis-Mendoza et al, and another by Beran et

al. Thus, while the sentence “A one-point increase in HbA1c was associated with an 18% higher risk of depression,” pertains to the study by Beran et al, the statement “and depression caused worse HbA1c outcomes” pertains to the study by Genis-Mendoza et al, who examined the relationship between hba1c and depression in three different groups.

Nevertheless, we have made the following changes to our writing to ensure that the reviews are incorporated appropriately. Results->Study Characteristics->Associations Identified->First Paragraph now reads as follows:

“A bidirectional longitudinal relationship between depressive symptoms and HbA1c levels in individuals with type 2 diabetes (T2DM) was identified [38]. Higher baseline depressive symptoms were associated with subsequent increases in HbA1c (partial r = 0.07, 95% CI: 0.03–0.12), while higher baseline HbA1c was linked to an 18% increased risk of developing depression (OR = 1.18, 95% CI: 1.12–1.25) [38]. Complementing these findings, another study showed that individuals with T2DM and depression had significantly higher HbA1c levels compared to those without depression (Cohen’s d = 0.18, 95% CI: 0.12–0.29)39. This association remained consistent in subgroups taking hypoglycemic medications (d = 0.20), with less than 10 years of disease duration (d = 0.17), and with diabetes-related complications (d = 0.17). Together, these findings underscore a complex, bidirectional relationship between glycemic control and depression in T2DM (Figure 1-Panel A).”

In addition Results->Study Characteristics->Associations Identified->Second Paragraph->Third Sentence now reads as follows:

“Additionally, both short and long sleep durations were associated with elevated HbA1c levels-0.23% and 0.13% [37], respectively suggesting a U-shaped relationship between sleep duration and glycaemic control [37].”

To further differentiate between the systematic review and umbrella review, the following paragraph was included at end of the introduction section:

“To contrast systematic and umbrella reviews, while systematic reviews are rigorous syntheses of primary research studies focused on a specific question, umbrella reviews synthesise findings from multiple systematic reviews and meta-analyses, offering a high-level summary across related topics. While systematic reviews examine individual studies, umbrella reviews evaluate the consistency, gaps, and convergence of evidence across existing reviews. This distinction is especially important in our study, which aims to assess the broader landscape of observational evidence on HbA1c and health risk behaviours, rather than re-analysing primary data. As such, umbrella reviews often adopt narrower search terms than systematic reviews to maintain specificity and avoid redundancy, as they synthesise existing reviews rather than primary studies. A broader search may increase sensitivity but risks including overlapping or less relevant reviews, complicating synthesis[32].”

Comment: 10) For A1c units, did all report A1c as % vs mmol/l (SI units)? Which unit for A1c is being used in Figure 1?

Response: Thank you for noticing this oversight. The reporting is in %. To clarify this, we have included in: Study Design and Methods->Data collection process the following sentence “Also, unless otherwise stated all report HbA1c as percentage (% units).”

To clarify Figure 1, we have modified the title as follows. “Relationship between depression, sleep, smoking and HbA1c. Reporting unit is in %.”

Comment: 11) Does this refer to CCA metric of less than 2% noted in results? Can you provide more details beyond brief sentence/reference of what a CCA metric is?

Response: We would once again like to thank the reviewer for allowing us to better present our work. To address this comment, we have included the following sentences in Study Design and Methods Study selection

"The CCA is a quantitative measure used to assess the degree of overlap among primary studies included in multiple systematic reviews. It is particularly useful in umbrella reviews or overviews of reviews to evaluate redundancy and ensure that findings are not disproportionately influenced by the same underlying studies. The CCA is calculated by comparing the number of repeated studies across reviews to the total number of unique studies, adjusted for the number of reviews. A CCA value of 0–5% indicates slight overlap, 6–10% moderate, 11–15% high, and >15% very high overlap [31 32]."

Comment: 12) In discussion - can you provide a reference for this sentence: Thus, for instance a review of analyses from the large longitudinal Nurses' Health Study in the USA found poor agreement between results from the study and RCT

Response: We are grateful for allowing us the opportunity for clarification. The sentence now reads as follows:

"Thus, for instance, a systematic review of analyses from the large longitudinal Nurses' Health Study in the USA found poor concordance between the results of observational analyses and those from RCTs, with agreement observed in only about 25% of cases [52]."

The reference provided is: Kar D, Gillies C, Nath M, et al. Association of smoking and cardiometabolic parameters with albuminuria in people with type 2 diabetes mellitus: a systematic review and meta-analysis. Acta diabetologica 2019;56:839-50.

Comment: 13) Authors note all data included in paper but was there any additional data extracted across the articles that was used to inform results/discussion? Relates to systematic/umbrella review clarification

Response: Thank you very much. Since this is an umbrella review, all extracted information from the relevant systematic reviews were presented in the tables/results/discussion. No additional data was extracted.

Reviewer 2

Comment: Thank you for the opportunity to review your work. My comments focus on the search methods and reporting. The umbrella review appears to be novel, with an extensive rationale provided for why an overview of reviews of observational studies on this topic is needed. Registration of the protocol in PROSPERO is another strength.

Response: Thank you very much for these supportive comments.

Comment: 1. I think that the search terms are not comprehensive enough to achieve the review's goals. Only the PubMed search is reported, so I can't assess the strategy used in other databases. In the PubMed search, most concepts are searched only in title and abstract fields; a few MeSH terms are included, but concerningly, not the MeSH term Glycated Hemoglobin. One of the free text terms reported for HbA1c appears to be a typo: "Hemoglobine A1c"[tiab] returns only 4 results in PubMed. The variant spelling haemoglobin is not included.

Response: We would like to thank the reviewer for allowing us to better clarify our work. We have corrected the typo. We would also like the reviewer to note that the last search term in the text string is "Glycated Hemoglobin", therefore we have included the MeSH term Glycated Hemoglobin. Also, the syntax of the pubmed search is provided, but the same search terms were used in other databases as well. The other search strings are provided in Appendix A. To clarify this, we have included the following sentence in Study design and methods->Information source and search strategy->Second paragraph->Last sentence

"While the search syntax provided below is for the PubMed search, the same search strategy was used for other databases as well (Appendix A). The search is as follows:"

While ideally both the American and British variants of the search term could be used, the American term appears to be used more substantially often in the literature. Thus for example while a pubmed search in the last ten years of "hemoglobin" in the literature gives us 8,304 results, searching for "haemoglobin" produces only 1,549 results. Nevertheless, to acknowledge this as a limitation we have included the following sentence in the limitations section:

"Spelling variations such as "haemoglobin" may also be incorporated"

It is also important to note that there are fundamental differences between a systematic review and an umbrella review such a this. An umbrella review follows a simpler search strategy than a systematic review. Thus according to experts on this topic (1) "Search strategies for reviews of reviews will be simpler than those for systematic reviews of single studies as the included reviews will already have undertaken these searches (pg 86)…." To distinguish between systematic and umbrella reviews we have included the following paragraph towards the end of last paragraph before "Aims of Study"->

"To contrast systematic and umbrella reviews, while systematic reviews are rigorous syntheses of primary research studies focused on a specific question, umbrella reviews synthesise findings from multiple systematic reviews and meta-analyses, offering a high-level summary across related topics. While systematic reviews examine individual studies, umbrella reviews evaluate the consistency, gaps, and convergence of evidence across existing reviews. This distinction is especially important in our study, which aims to assess the broader landscape of observational evidence on HbA1c and health risk behaviours, rather than re-analysing primary data. As such, umbrella reviews often adopt narrower search terms than systematic reviews to maintain specificity and avoid redundancy, as they synthesise existing reviews rather than primary studies. A broader search may increase sensitivity but risks including overlapping or less relevant reviews, complicating synthesis[32]"

Also, in an umbrella review of systematic reviews (unlike a systematic review) we expect the key findings of the specific systematic review to be highlighted in the title and/or the abstract. As such it is not uncommon for umbrella reviews to limit the search to Title and Abstract. For example, see:

Favril, L., Rich, J. D., Hard, J., & Fazel, S. (2024). Mental and physical health morbidity among people in prisons: an umbrella review. ***The lancet public health***, *9*(4), e250-e260.

Nevertheless, to acknowledge this fact we have modified the limitations section – Discussion->Last Paragraph->First Sentence as follows:

"While this review covers a comprehensive and unique set of search terms, being one of the first reviews of its kind, there is scope for further analysis and expansion, such as the expansion of the initial search from beyond title and abstract."

Comment: Additionally, the terms for the 6 key health risk factors are very restricted. For example, the term smoking is searched in title and abstract, but not tobacco or nicotine; diet, but not dietary or diets or other diet-related terms (which could miss key reviews on for instance, sugar intake); physical activity and sedentarism, but not exercise or sedentary time/behaviour. There are terms for anxiety, depression, mood disorder, stress and distress, but the term "mental health" is not included. The lack of MeSH terms for most of the health risk behaviours may have led to missing relevant reviews, since some articles lack abstracts. There could have been more use of the * after terms to find various word endings, such as after mood disorder* or depressive symptom*.

Response: Thank you for allowing us to clarify this. Our responses to each of the issues are provided below. The key issue is that being an umbrella review our aim was to synthesise existing systematic reviews rather than conduct an exhaustive primary-level search. Therefore, we prioritised a focused and conceptually coherent search strategy to ensure feasibility, manageability, and alignment with our predefined framework. Overly broad search terms would have significantly increased the volume of irrelevant results, potentially diluting the specificity and clarity of our synthesis. As such, we have included the following sentence to the end of the Aims of the Study section->

"Our overall aim was to synthesise existing systematic reviews and meta-analyses rather than conduct an exhaustive primary-level search. Therefore, we prioritised a focused and conceptually coherent search strategy to ensure feasibility, manageability, and alignment with our predefined framework."

The following paragraph has also been added to the end of the introduction section:

"To contrast while systematic reviews are rigorous syntheses of primary research studies focused on a specific question, umbrella reviews synthesise findings from multiple systematic reviews and meta-analyses, offering a high-level summary across related topics. While systematic reviews examine individual studies, umbrella reviews evaluate the consistency, gaps, and convergence of evidence across existing reviews. This distinction is especially important in our study, which aims to assess the broader landscape of observational evidence on HbA1c and health risk behaviours, rather than re-analyzing primary data."

Use of the eVITAL Taxonomy:
The selection of health risk behaviour (HRB) terms was informed by the **eVITAL taxonomy** (Salvador-Carulla et al., 2012; 2013), which provides a structured framework for categorizing lifestyle and behavioural health factors. This taxonomy was used to ensure conceptual consistency across exposures and to facilitate coding and synthesis. As such, terms like "diet," "sleep," and "physical activity" were chosen to align with specific eVITAL categories. To better highlight the salience of the eVITAL framework we have included the following sentences to the Aims of the Study section:

"The previously published eVITAL taxonomy provides a structured and validated framework for categorizing lifestyle mental health, and behavioural health domains, ensuring conceptual clarity and consistency across studies. Its use enhances the reproducibility and comprehensiveness of search strategies by aligning terms with well-defined behavioural constructs [25 29].

Inclusion of MeSH Terms:
The search strategy did include MeSH terms where appropriate. For example, "depressive disorder" was searched using the MeSH term, and other terms such as "depress*" and "mood disorder" were included to capture variations. We agree that additional wildcard use (e.g., "diet*") could enhance retrieval and will consider this in future updates or replications of the review.

Search Scope and Feasibility:
Given the scope of this umbrella review and the need to balance sensitivity with specificity, we focused on terms most directly aligned with our conceptual framework. While, we acknowledge that terms such as "mental health" could have captured additional relevant studies, this would have generated a very large set of results and reduced the specificity of the results, hence we have selected the two mental health issues found to be the most salient to hba1c. We have included the following reference to the sentence "We chose six key health risk factors, - mental health conditions, sleep characteristics, diet, physical activity, alcohol and smoking that, in separate studies, have been found to be associated with HbA1c" in "Aims of the Study"->Second sentence:

"Sun, N., Lou, P., Shang, Y., Zhang, P., Wang, J., Chang, G., & Shi, C. (2016). Prevalence and determinants of depressive and anxiety symptoms in adults with type 2 diabetes in China: a cross-sectional study. *BMJ open*, *6*(8), e012540."

Comment: An additional valuable field that could have been searched for free text is author-provided keywords. The text word [tw] field code in PubMed searches for matches in title, abstract, author-provided keywords, and MeSH fields. Please note though that it is best practice to also search MeSH terms in addition to terms in [tw], as using the MeSH will expand your search to include narrower, related MeSH terms. As an example, a search for Diet[MeSH] will include all the terms listed at the bottom of this page - https://www.ncbi.nlm.nih.gov/mesh/68004032 - so could find a review on intermittent fasting's impact on HbA1c where the term "diet" might not be used in the title or abstract.

Response: Thank you. As noted in the previous comment, we have indeed included MeSH terms in our search. We would also like to thank the reviewer for recommending the use of the term using [tw] for MeSH. However, according to research (see, Salvador-Oliván, J. A., Marco-Cuenca, G., & Arquero-Avilés, R. (2019). Errors in search strategies used in systematic reviews and their effects on information retrieval. *Journal of the Medical*

Library Association: JMLA, *107*(2), 210). Using, [tw] for MeSH terms can reduce recall because it does not explode the term. While we also agree that the use of [MeSH] may have increased retrieval, it also has the downside of not searching non-indexed papers. Nevertheless, to accommodate for this issue in our paper, we have included the following sentence in the discussion limitation section, Discussion-> fifth paragraph->third sentence now reads as follows

"Another possible scope for expansion is the use of the [tw] term in search or the including of [MeSH], which when used with caution, can support a more comprehensive review[51]."

Comment: A very comprehensive search might include a search only for HbA1c (including the MeSH term for it) plus the systematic review filter, then screening for reviews which discuss health risk behaviours. The screening burden would certainly be higher using this approach, but it runs less risk of omitting relevant reviews.

Response: We would like to thank the reviewer for this insightful comment. We agree that a broader search could have yielded more reviews for inclusion. The decision of an optimal trade-off between parsimony and comprehensiveness is difficult. Especially since this is an umbrella review and not a systematic review that seeks to comprehensively review primary literature as pointed out at the beginning of the responses to Reviewer-2. To acknowledge this shortcoming in our study we have included the following sentences in the limitations for our Discussion->Last Paragraph->Second sentence now reads as follows:

"A very comprehensive search might include a search only for HbA1c (including the MeSH term for it) plus the systematic review filter, then screening for reviews which discuss health risk behaviours reducing risk of omitting relevant reviews"

Comment: 2. Searches could be reported more clearly. Item 7 in the PRIOR checklist says "Present the full search strategies for all databases, registers and websites, such that they could be reproduced. Describe any search filters and limits applied." Please add to the supplement the exact search terms used in Scopus, Web of Science, and PsycINFO-PsycArticles. Further, for Web of Science, please list which databases were included in your search (such as Science Citation Index, Social Sciences Citation Index). Databases in Web of Science can vary a lot from one institution to another. Also, please indicate which platform PsycInfo/PsycArticles were searched through - EBSCO? Ovid?

For greater transparency, I recommend reporting the terms used in the Systematic[sb] filter in PubMed, as this filter may change over time or be removed. The terms included as of May 9, 2025 are:

(((systematic review[ti] OR systematic literature review[ti] OR systematic scoping review[ti] OR systematic narrative review[ti] OR systematic qualitative review[ti] OR systematic evidence review[ti] OR systematic quantitative review[ti] OR systematic meta-review[ti] OR systematic critical review[ti] OR systematic mixed studies review[ti] OR systematic mapping review[ti] OR systematic cochrane review[ti] OR systematic search and review[ti] OR systematic integrative review[ti]) NOT comment[pt] NOT (protocol[ti] OR protocols[ti])) NOT MEDLINE [subset]) OR (Cochrane Database Syst Rev[ta] AND review[pt]) OR systematic review[pt]

(https://www.nlm.nih.gov/bsd/pubmed_subsets/sysreviews_strategy.html)

Note that the filter does not effectively find scoping reviews, rapid reviews, or some meta-analyses (those which may not mention the term systematic in the title or be tagged with the publication type). While you were only specifically looking for systematic reviews or meta-analyses, I noted that one integrative review was included in your umbrella review.

Response: Thank you very much for this comment and we have addressed the various issues as described below:

We have included in Appendix A all search strings used in the various databases

To better indicate the specific databases we searched, we have included in Study Design and Methods->Information source and search strategy->First Sentence "The search was implemented on Pubmed, Scopus, Web of Science (List of databases provided in appendix A) and PsycINFO-PsychArticles (EBSCO) from…"

We have included the search terms included within "sb" in Appendix A and have included in Study Design and Methods->Information source and search strategy->Second Paragraph->Last Sentence

"Terms included in the sb filter in pubmed are provided in Appendix A"

Regarding the integrative review in this search, it was probably in the search results and selected because the integrative review was preceded by a systematic review search strategy. To better clarify this,we have changed our writing as follows:

Results->Study Characteristics->Second Paragraph->First Sentence now reads as follows:

Six reviews implemented a meta-analysis while one review implemented only a systematic review [33] and another, Zhu, et al. [34] implemented an integrative review following a systematic review of the literature.

Comment: The review would be strengthened with an updated search to include any studies published or added to databases since Sept 2023. I strongly recommend updating the search, and also expanding the search to include Epistemonikos, a specialty database of systematic reviews: https://www.epistemonikos.org/ I would advocate using a simple search in Epistemonikos, such as just HbA1c and its synonyms, and screening the results.

Response: Thank you. We have expanded the search to until June 2025. We have also included the Epistemonikos database in our search. All results have thus been updated, though no new reviews satisfied the selection criteria for synthesis. As discussed earlier, a reasonable tradeoff between specificity and sensitivity is desirable, as is understanding the differences in scope of an umbrella review versus a systematic review and hence we have retained the original search string.

Comment: Also, I wonder if you could explain why the search was limited to 2013-present. Given that there seem to be few reviews covering only observational studies, why not look back farther?

Response: Thank you. The original review went back a decade/10 years. This was to ensure that there is currency in what we report. Our review now goes back approximately 12 years. We have included the following sentences in our text:

"The search was implemented on Pubmed, Scopus, Web of Science and PsycINFO-PsychArticles from January 2013 to 30 April 2025. This corresponds to approximately a 12-year time window, allowing us to assess relatively contemporary literature on the topic."

Comment: I have some more minor comments for your consideration:

Response: Thank you. They are addressed below.

Comment: 4. You state that the search was performed by 2 researchers. This isn't necessary, unlike other review steps like screening, data extraction and quality assessment that benefit from two people's involvement in order to limit bias. While two people or the whole team might have been involved in developing the search, it's sufficient for one person to run the final search and export results to the tool you are using for screening.

Response: Thank you for this comment. We shall keep this in mind during future reviews to optimise our resources.

Comment: 5. Could you please report how deduplication was done? Eg Covidence, Endnote, some other process? This is one of the items noted in the PRISMA-S extension, which may be of interest: https://doi.org/10.1186/s13643-020-01542-z

Response: Thank you. We have included a sentence in Results->Study selection-> "Deduplication was done in EndNote[33]"

Comment: 6. While the search was not limited to English, I noticed in the PRISMA flow diagram 1 non-English study was excluded. I suggest stating the restriction to English in your eligibility criteria and in the limitations. Also, Table 1 appears to be missing from the manuscript.

Response: Thank you for pointing out these oversights.

The following sentence has been included in limitations "Finally, including studies not written in English also provides an opportunity for expansion." Also, the following sentence has been included in Results->Study Selection "The excluded articles included reviews that were not exclusively based on observational studies, and one that was not in English."

Table 1 was providing redundant information and was removed; however, the table numbers (Table 2->Table 1) had not been previously updated. This has now been done.

Comment: 7. On page 5 you mention the "Cochrane Pyramid" - this term wasn't familiar to me, it might be clearer to call it the EBM pyramid? I know what you're referring to, but I'm unclear that it has its origin with Cochrane or is generally known by that name.

Response: Thank you. We have replaced Cochrane pyramid with "evidence-based medicine pyramid"

Comment: 8. Perhaps I missed it but I did not find a completed PRIOR checklist in the supplementary materials.

Response: Thank you again for observing this oversight. The checklist was not previously uploaded and has now been included in the submission.

References

1. Ortega A, Lopez-Briz E, Fraga-Fuentes MD. From Qualitative Reviews to Umbrella Reviews. In: Biondi-Zoccai G, editor. Umbrella Reviews: Evidence Synthesis with Overviews of Reviews and Meta-Epidemiologic Studies. Cham: Springer International Publishing; 2016. p. 21-41.
2. Groenwold RH. Trial emulation and real-world evidence. JAMA Network Open. 2021;4(3):e213845-e.
3. Patsopoulos NA. A pragmatic view on pragmatic trials. Dialogues in clinical neuroscience. 2011;13(2):217-24.

VERSION 2 - REVIEW

**Reviewer**	**1**
**Name**	**Dorans, Kirsten S**
**Affiliation**	**Tulane University**
**Date**	**21-Jul-2025**
**COI**	

Note to editors: recommend re-review by someone with expertise in systematic reviews and umbrella reviews regarding if the searches/methods described are appropriate.

Thank you for this thorough review in response to author comments.

Additional comments:

1) Authors should still note in the introduction and again in discussion/limitations subsection that there are potential limitations of confounding, reverse causation, other potential bias in the findings from this umbrella review due to it focusing on observational studies. The core limitations of observational studies was still not clearly and fully addressed in the revisions.

2) For Figure 2 and discussion of Figure 2 - regarding the directionality, one of the challenges of doing observational studies is potential for reverse causation. Can you address this in the discussion and also in describing Figure 2 and also in the legend (make note that arrows going in one direction for those that are cross-sectional)?

Minor:

1) Page 13 - line 50 - please include units for HbA1c (between smokers and ex-smokers)

2). Page 14 lines 53-54- please provide references. More generally, do you find many more studies that are randomized and can you report on numbers that you found that are randomized (rather than only stating that many more are randomized)?

3) Results - do you mean to state "40 were excluded because they were not meta-analysis, systematic reviews, or human studies)? (currently states 'not... non-human studies).

**Reviewer**	**2**
**Name**	**Ellis, Ursula**
**Affiliation**	**The University of British Columbia, Woodward Library**
**Date**	**21-Jul-2025**
**COI**	

Thank you for adding documentation of the other database searches, running a search of Epistemonikos, and updating the searches.

I am still concerned about the narrow scope of the search. I think it's important to broaden the search terms related to the health risk behaviours, and include the MeSH term Glycated Hemoglobin, as well as keyword spelling variant haemoglobin A1c.

As you've found, it's difficult to find reviews that exclusively include observational studies, so I think it's warranted to use a more sensitive search to ensure you're finding everything. As Salvidar-Olivan et al (2019) show in the study you cite, by far the most common search errors in knowledge synthesis search strategies involve missing terms - both keywords and MeSH - resulting in potentially missed studies.

Your statement that umbrella reviews "often adopt narrower search terms than systematic reviews to maintain specificity and avoid redundancy" doesn't align with my experience. Authoritative guidance documents for umbrella reviews or overviews of reviews, such as the JBI Manual for Evidence Synthesis and the Cochrane Handbook, emphasize the importance of a comprehensive search strategy. It's certainly encouraged to limit the search with a systematic review-focused filter like you've done. However, I don't think it's wise to search only titles and abstracts for other key concepts, especially given the variability in how the health behaviours you've selected may be described.

You mention on page 6 of your response to reviewers that "A number of terms in our search are MeSH Terms as follows:"

Sleep Disorders

Sleep Wake Disorders

Sleep Deprivation

Insomnia

Obstructive Sleep Apnea

Anxiety

Mood Disorders

Stress, Psychological

Psychological Distress

Depressive Disorder

Dysthymic Disorder

Smoking

Diet

However, on page 9 of your revised manuscript the search is reported as:

Systematic[sb] AND (("Sleep disorder*"[Title/Abstract] OR "Sleep wake disorder*"[Title/Abstract] OR "Sleep deprivation"[Title/Abstract] OR "Sleep arousal"[Title/Abstract] OR "Insomnia"[Title/Abstract] OR "Obstructive sleep apnea"[Title/Abstract] OR Anxiety[Title/Abstract] OR "Mood disorder"[Title/Abstract] OR Stress[Title/Abstract] OR "Psychological distress"[Title/Abstract] OR “depressive disorder”[MeSH Terms] OR depress*[Title/Abstract] OR "Depressive disorder"[Title/Abstract] OR "Depressive symptom"[Title/Abstract] OR "Dysthymic disorder"[Title/Abstract] OR "alcohol"[Title/Abstract] OR "smoking"[Title/Abstract] OR "diet"[Title/Abstract] OR "Physical activity"[Title/Abstract] OR sedentarism"[Title/Abstract]) AND (HbA1c[Title/Abstract] OR "Hemoglobin A1C"[Title/Abstract] OR "Glycated Hemoglobin"[Title/Abstract]))

The only MeSH term present in this reported search is "depressive disorder." Searching Title/Abstract does not include a search of the MeSH field of the database record. Also, note that there is a typo in the search terms - a missing " before sedentarism - causing an error when rerunning the search.

You mention that you corrected the typo of "Hemoglobine A1C" for the PubMed search, yet it appears in Appendix 1 in the Scopus, Web of Science, and PsycInfo searches - when you ran the search update, did you use this spelling? Only searching HbA1c, hemoglobine A1c, and glycated hemoglobin in titles and abstracts in these databases might miss articles using only the phrase "hemoglobin A1c."

Also, the Web of Science search syntax looks incorrect, as though the Scopus search terms have been copied here. It's not possible to search title and abstract as a combined field in Web of Science. The format should look like: TI=(search terms) OR AB=(search terms); and the review limit would look like: Review Article (Document Types).

I think it would be feasible to run an additional search update implementing some of the recommended changes - at a minimum, including the MeSH term Glycated Hemoglobin in the PubMed search, which reviewer 1 also expressed concerns about - without resulting in an overwhelming additional number to screen. Completing a more extensive search would increase the credibility of your findings.

A few more minor comments:

It's best practice to document the total number of results found in each database in the Appendix and/or PRISMA flow diagram, per the PRISMA-S extension, item 15: https://doi.org/10.1186/s13643-020-0154.

This supports reproducibility, as reviewers or other readers can rerun your search and compare the number of results.

PRISMA-S also recommends documenting that a search update was done, with some suggested wording in item 12.

Thanks for clarifying that a librarian was involved in developing the search. Will you consider acknowledging them by name in the published manuscript (with their consent)?

VERSION 2 - AUTHOR RESPONSE

Reviewer: 2

Ms. Ursula Ellis, The University of British Columbia

Comments to the Author:

Comment	Response
Thank you for adding documentation of the other database searches, running a search of Epistemonikos, and updating the searches.	Thank you for your support.
I am still concerned about the narrow scope of the search. I think it's important to broaden the search terms related to the health risk behaviours, and include the MeSH term Glycated Hemoglobin, as well as keyword spelling variant haemoglobin A1c.	Thank you very much for these comments. The tradeoff between being comprehensive and parsimonious is difficult. We have tried to ensure that our search is both parsimonious and reproducible. Hence, the use of the eVITAL health risk behaviour terms which provides a structured and validated framework for categorizing lifestyle and behavioural health domains. In addition, please see our response to the later comment regarding MeSH terms. The fact that our findings are very convergent with what we know about the topic from other studies in the area (with evidence from RCTs) shows that our study is credible and has a reasonable scope, and achieves what Salvador-Olivan calls "an equilibrium between recall and precision".

As you've found, it's difficult to find reviews that exclusively include observational studies, so I think it's warranted to use a more sensitive search to ensure you're finding everything. As Salvidar-Olivan et al (2019) show in the study you cite, by far the most common search errors in knowledge synthesis search strategies involve missing terms - both keywords and MeSH - resulting in potentially missed studies	Thank you very much for this insightful comment. While it is true that we find very few observational studies, we do believe that we have found a parsimonious set of systematic reviews, and further expansion of the search would have been unnecessary. Indeed the paper by Salvador-Olivan that the reviewer states begins (in the second paragraph) with the following statement "The ideal result has high rates of both recall and precision, although this goal is difficult to achieve due to the tradeoff relationship that exists between the two. Hence, it is necessary to find an equilibrium between them".
Your statement that umbrella reviews "often adopt narrower search terms than systematic reviews to maintain specificity and avoid redundancy" doesn't align with my experience. Authoritative guidance documents for umbrella reviews or overviews of reviews, such as the JBI Manual for Evidence Synthesis and the Cochrane Handbook, emphasize the importance of a comprehensive search strategy. It's certainly encouraged to limit the search with a systematic review-focused filter like you've done. However, I don't think it's wise to search only titles and abstracts for other key concepts, especially given the variability in how the health behaviours you've selected may be described.	Thank you for these insightful comments. Our approach of focusing only on title and abstracts is not uncommon in the literature. Examples include umbrella reviews from mental health[1], carceralogy[2], criminology[3] and exercise science [4]. We have included the following sentence in the limitations section "While our study utilises a title and abstract search following some other umbrella reviews [62] [63] ...." it may also be beneficial to implement the initial search on full text,"

You mention on page 6 of your response to reviewers that "A number of terms in our search are MeSH Terms as follows:"<br><br>Sleep Disorders<br>Sleep Wake Disorders Sleep<br>Deprivation Insomnia<br>Obstructive Sleep Apnea<br>Anxiety<br>Mood Disorders<br>Stress, Psychological<br>Psychological Distress<br>Depressive Disorder<br>Dysthymic Disorder<br>Smoking<br>Diet<br><br>However, on page 9 of your revised manuscript the search is reported as:<br><br>Systematic[sb] AND (("Sleep disorder*"[Title/Abstract] OR "Sleep wake disorder*"[Title/Abstract] OR "Sleep deprivation"[Title/Abstract]	Thank you. We were of the view that the reviewer was asking about the use of MeSH Terms rather than a search of MeSH indices in the database. We are aware that including MeSH terms in the title/abstract does not search the database itself. According to Leblanc, V., Hamroun, A., Bentegeac, R., Le Guellec, B., Lenain, R., & Chazard, E. (2024). Added Value of Medical Subject Headings Terms in Search Strategies of Systematic Reviews: Comparative Study. *Journal of medical Internet research*, *26*, e53781; addition of MeSH terms did not reduce sensitivity in the majority (59%) of studies. A mix of MeSH terms is sometimes recommended and we have one. Hence we believe that our study is parsimonious and comprehensive to the extent necessary.<br>Thank you very much for this suggestion. According to Leblanc, V., Hamroun, A., Bentegeac, R., Le Guellec, B., Lenain, R., & Chazard, E. (2024). Added Value of Medical Subject Headings Terms in Search Strategies of Systematic Reviews: Comparative Study. Journal of medical Internet research, 26, e53781; addition of MeSH terms did not reduce sensitivity in the majority (59%) of studies. As such, especially given that our findings support what is already known in the literature, but from other sources of information (syntheses of RCT studies), we believe that our findings are credible. We have now modified the first part of the limitations section (Discussion->Last paragraph), which now reads as follows:

OR "Sleep arousal"[Title/Abstract] OR "Insomnia"[Title/Abstract] OR "Obstructive sleep apnea"[Title/Abstract] OR Anxiety[Title/Abstract] OR "Mood disorder"[Title/Abstract] OR Stress[Title/Abstract] OR "Psychological distress"[Title/Abstract] OR "depressive disorder"[MeSH Terms] OR depress*[Title/Abstract] OR "Depressive disorder"[Title/Abstract] OR "Depressive symptom"[Title/Abstract] OR "Dysthymic disorder"[Title/Abstract] OR "alcohol"[Title/Abstract] OR "smoking"[Title/Abstract] OR "diet"[Title/Abstract] OR "Physical activity"[Title/Abstract] OR sedentarism"[Title/Abstract]) AND (HbA1c[Title/Abstract] OR "Hemoglobin A1C"[Title/Abstract] OR "Glycated Hemoglobin"[Title/Abstract]))<br><br>The only MeSH term present in this reported search is "depressive disorder." Searching Title/Abstract does not include a search of the MeSH field of the database record. Also, note that there is a typo in the search terms - a missing " before sedentarism - causing an error when rerunning the search.	"While this review covers a comprehensive and unique set of search terms, being one of the first reviews of its kind, there is scope for further analysis and expansion, such as the expansion of the initial search from beyond title and abstract. A very comprehensive search might include a search only for HbA1c (including the MeSH term for it) plus the systematic review filter, then screening for reviews which discuss HRBs reducing risk of omitting relevant reviews. Another possible scope for expansion is the use of the [tw] term in search or the including of [MeSH], which when used with caution, can support a more comprehensive review[59]. While our study utilises a title and abstract search following some other umbrella reviews [62 63], it may also be beneficial to implement the initial search on full text, although the addition of MeSH terms did not reduce sensitivity in the majority (59%) of studies [64]"<br><br>The missing " before the sedentarism " is present in the search, hence there is no error, we have now corrected the manuscript to reflect this.
You mention that you corrected the typo of "Hemoglobine A1C" for the PubMed search, yet it appears in Appendix 1 in the Scopus, Web of Science, and PsycInfo searches - when you ran the search update, did	The erroneous spelling was saved into MS Word's dictionary resulting in erroneous corrections. This has now bee corrected.

you use this spelling? Only searching HbA1c, hemoglobine A1c, and glycated hemoglobin in titles and abstracts in these databases might miss articles using only the phrase "hemoglobin A1c."	
Also, the Web of Science search syntax looks incorrect, as though the Scopus search terms have been copied here. It's not possible to search title and abstract as a combined field in Web of Science. The format should look like: TI=(search terms) OR AB=(search terms); and the review limit would look like: Review Article (Document Types).	Thanks. This has now been corrected.
I think it would be feasible to run an additional search update implementing some of the recommended changes - at a minimum, including the MeSH term Glycated Hemoglobin in the PubMed search, which reviewer 1 also expressed concerns about - without resulting in an overwhelming additional number to screen. Completing a more extensive search would increase the credibility of your findings.	Thank you very much for this suggestion. According to Leblanc, V., Hamroun, A., Bentegeac, R., Le Guellec, B., Lenain, R., & Chazard, E. (2024). Added Value of Medical Subject Headings Terms in Search Strategies of Systematic Reviews: Comparative Study. *Journal of medical Internet research*, *26*, e53781; addition of MeSH terms did not reduce sensitivity in the majority (59%) of studies. As such, especially given that our findings support what is already known in the literature, but from other sources of information (syntheses of RCT studies), we believe that our findings are credible. We have now modified the first part of the

	limitations section (Discussion->Last paragraph), which now reads as follows:<br>"While this review covers a comprehensive and unique set of search terms, being one of the first reviews of its kind, there is scope for further analysis and expansion, such as the expansion of the initial search from beyond title and abstract. A very comprehensive search might include a search only for HbA1c (including the MeSH term for it) plus the systematic review filter, then screening for reviews which discuss HRBs reducing risk of omitting relevant reviews. Another possible scope for expansion is the use of the [tw] term in search or the including of [MeSH], which when used with caution, can support a more comprehensive review[59]. While our study utilises a title and abstract search following some other umbrella reviews [62 63], it may also be beneficial to implement the initial search on full text, although the addition of MeSH terms did not reduce sensitivity in the majority (59%) of studies [64]"
It's best practice to document the total number of results found in each database in the Appendix and/or PRISMA flow diagram, per the PRISMA-S extension, item 15: https://doi.org/10.1186/s13643-020-0154.<br>This supports reproducibility, as reviewers or other readers can rerun	We are grateful for these comments. Please note that the doi link provided by the reviewer does not seem to be working.<br>We looked up the guide sheet for PRISMA-S (which is for scoping reviews and not umbrella reviews) and item 15 appears to be about

your search and compare the number of results.<br><br>PRISMA-S also recommends documenting that a search update was done, with some suggested wording in item 12.	“characteristics and sources of evidence” which is unrelated to the comment.<br>Neverhtless, following the reviewers suggestions we have included the following sentence in Study Design and Methods->Information Source and Search Strategy->Third Sentence:<br><br>“While the original search was until September 2024, and the search was later updated to June 2025.”<br>Thank you for suggesting that we include the total numbers for each database. The PRISMA flow diagrams instruct “*Consider, if feasible to do so, reporting the number of records identified from each database or register searched (rather than the total number across all databases/registers)”; hence it is a suggestion. Unfortunately, in our case this is not feasible.
Thanks for clarifying that a librarian was involved in developing the search. Will you consider acknowledging them by name in the published manuscript (with their consent)?	Thank you. We are now acknowledging the librarian in the paper.

Reviewer: 1

Dr. Kirsten S Dorans, Tulane University

Comments to the Author:

**Comment**	**Response**
Note to editors: recommend re-review by someone with expertise in systematic reviews and umbrella reviews regarding if the searches/methods described are appropriate. Thank you for this thorough review in response to author comments.	Thank you.
1) Authors should still note in the introduction and again in discussion/limitations subsection that there are potential limitations of confounding, reverse causation, other potential bias in the findings from this umbrella review due to it focusing on observational studies. The core limitations of observational studies was still not clearly and fully addressed in the revisions.	Thank you for this insightful comment. While it is true that observational studies may have issues confounding, reverse causation, other potential bias, it must also be noted that our findings agreed with what has been found in syntheses of RCTs on the similar topic (and this has been discussed in the paper). As such, while a perhaps a pertinent problem of observational studies in general, the findings from our review does not greatly diverge from the extant "causal" or more "rigorous" literature using RCT or similar designs. Nevertheless to acknowledge the existing weaknesses of observational studies in general, we have made the following changes:

	We have modified Introduction->Fifth paragraph->Third sentence to "While observational studies may have issues with confounding, reverse causation, other potential bias, when triangulated with other forms of knowledge, this can inform collective decision-making through consilience to drive health policy and research….."<br><br>In addition, the last paragraph of discussion, which discusses limitations now reads as follows (last two sentences):<br>"While there is potential for limitations of confounding, reverse causation, other potential bias in the findings from this umbrella review due to it focusing on observational studies, the results from this study largely agree with what has been found in other reviews that focus on RCTs. This review thus provides convergent and consilient evidence on the drivers correlates of HbA1c."
2) For Figure 2 and discussion of Figure 2 - regarding the directionality, one of the challenges of doing observational studies is potential for reverse causation. Can you address this in the discussion and also in describing Figure 2 and also in the legend (make note that arrows going in one direction for those that are cross-sectional)?	Thank you for this important observation. It is true that some observational studies may have issues with reverse causation, and this may be reflected in a study synthesis. Some of this is covered in the last section. Also our synthesis agrees with what has been found in synthesis of RCTs. While we acknowledge the importance of this comment, we are leaving the figure (Figure 2) unchanged, since mentioning reverse

	causation here is likely to confuse the reader, instead relying on an acknowledgement of the same in introduction and limitations.
Minor:<br>1) Page 13 - line 50 - please include units for HbA1c (between smokers and ex-smokers)	Thank you. To prevent repetition of units throughout the paper, we have clarified the units used in the paper at the very outset. Study Design and Methods->Data Collection process->First Paragraph-> Last sentence reads as follows "Also, unless otherwise stated all studies report HbA1c as percentage (% units)."
2). Page 14 lines 53-54- please provide references. More generally, do you find many more studies that are randomized and can you report on numbers that you found that are randomized (rather than only stating that many more are randomized)?	Thank you very much. Results-> Further Study selection->First Paragraph->Third to last sentence now reads as follows: "Finally, eight reviews were included [4 40-46]"<br><br>Results-> Further Study selection->First Paragraph->Second to last sentence now reads as follows: "Of the 606 studies, 467 or 77% involved RCTs".
3) Results - do you mean to state "40 were excluded because they were not meta-analysis, systematic reviews, or human studies)? (currently states 'not... non-human studies).	Thank you very much for noticing this error. We have now re-written the sentence to clarify its meaning as follows "Of the remaining 654 records, 40 were excluded as they were either not meta-analyses or systematic reviews, or involved non-human studies"

References

1. Jackson JL, Apilado KP, Koehlmoos TP. A Review of Reviews Assessing Patient–Provider Racial and Ethnic Concordance in Mental Health. *Journal of Racial and Ethnic Health Disparities* 2025:1-19.
2. Favril L, Rich JD, Hard J, et al. Mental and physical health morbidity among people in prisons: an umbrella review. *The lancet public health* 2024;9(4):e250-e60.
3. van IJzendoorn MH, Bakermans-Kranenburg MJ, Coughlan B, et al. Annual research review: Umbrella synthesis of meta-analyses on child maltreatment antecedents and interventions: Differential susceptibility perspective on risk and resilience. *Journal of child psychology and psychiatry* 2020;61(3):272-90.
4. Abu-Omar K, Rütten A, Burlacu I, et al. The cost-effectiveness of physical activity interventions: a systematic review of reviews.

Preventive medicine reports 2017;8:72-78.
